# Development of novel robotic platforms for mechanical stress induction, and their effects on plant morphology, elements, and metabolism

**DOI:** 10.1038/s41598-021-02581-9

**Published:** 2021-12-13

**Authors:** Polina Kurtser, Victor Castro-Alves, Ajay Arunachalam, Viktor Sjöberg, Ulf Hanell, Tuulia Hyötyläinen, Henrik Andreasson

**Affiliations:** 1 Centre for Applied Autonomous Sensor Systems, Örebro University, 701 82 Örebro, Sweden; 2 Man-Technology-Environment Research Centre, Örebro University, 701 82 Örebro, Sweden

**Keywords:** Mechanical engineering, Plant stress responses

## Abstract

This research evaluates the effect on herbal crops of mechanical stress induced by two specially developed robotic platforms. The changes in plant morphology, metabolite profiles, and element content are evaluated in a series of three empirical experiments, conducted in greenhouse and CNC growing bed conditions, for the case of basil plant growth. Results show significant changes in morphological features, including shortening of overall stem length by up to 40% and inter-node distances by up to 80%, for plants treated with a robotic mechanical stress-induction protocol, compared to control groups. Treated plants showed a significant increase in element absorption, by 20–250% compared to controls, and changes in the metabolite profiles suggested an improvement in plants’ nutritional profiles. These results suggest that repetitive, robotic, mechanical stimuli could be potentially beneficial for plants’ nutritional and taste properties, and could be performed with no human intervention (and therefore labor cost). The changes in morphological aspects of the plant could potentially replace practices involving chemical treatment of the plants, leading to more sustainable crop production.

## Introduction

The research field of plant stress studies plants’ reactions to sub-optimal conditions, with effects on growth, crop yield, and resilience to environmental conditions^1^. While permanent damage or death are possible if the stress exceeds the plant’s tolerance limits, some effects can be beneficial to the commercial plant-grower and end-consumers. Examples include heat stress and ultraviolet (UV) light stress^2,3^, which are used to increase plants’ resilience to extreme temperatures. Stress-inducing protocols are applied by nurseries that grow seedlings for replanting to obtain more resilient plants, an effect that is of special importance due to climate changes. An increase in UV light has also been found to influence plant morphology and metabolism, leading to changes in crop quality^4^.

Methods to change plant morphology, with the goal of growing more visually appealing plants, are used by commercial growers in decorative varieties^5,6^. Some more recent research also aimed to alter plant morphology to smooth the introduction of automation^7^ and mass production^5,6^. Finally, plant compactness is of interest to futuristic farming paradigms, such as vertical and roof-top farming^8^, where the environment is increasingly space-limited. Regulation of plant morphology is often done in practice using growth regulators^9^, chemical substances which govern all the factors of development and growth within plants. Since these substances are often regulated as pesticides^10^, the search for more sustainable alternatives such as stress induction is necessary.

While the effects of stress on plant morphology and resilience have been widely explored, a slightly more unique direction in the exploration of plant stress deals with its effects on taste, metabolites and nutrient content^11,12^. Since consumers are interested in healthier food consumption, and according to reports^13^ would pay 30–40% more for a healthier food product, exploring how stress affects plant health and nutritional content, in both greenhouse plants and outdoor-grown crops, is not only of a scientific and public-health value, but includes a commercial incentive as well^14^.

Mechanically induced stress (MIS), which this study focuses on, is plant stress induced by mechanical stimuli. In plants grown in outdoor conditions, the mechanical stimuli naturally occur as aerial parts are moved, usually by wind, rain or animals^6^. Plants subject to mechanical stress exhibit both alterations in plant morphology and increased resilience to harsh environmental conditions^15,16^. Maybe the most dramatic example of such an effect is present in carnivorous plants, which modify their leaves into a closing trap within seconds, in response to contact by insects^15^.

The effect of mechanically induced stress can be observed visually due to the significant reduction in plant size (including stem and inter-node distances) and increased plant compactness^6,16^. Manual stimulations of the plant, of various kinds^5,6,15,16^, have been suggested to reproduce the effect in indoor conditions. Since the stimulation is manual the number of plants is often limited, and so is the ability to produce statistically significant results. Where the mechanical stress stimuli are automated, as required in commercial efforts (e.g., Refs.^5,16^) the ability to vary the treatment between plants is limited, since the automation often treats hundreds of pots in exactly the same manner with a single mechanical tool. This limits the ability to vary growing protocols and species, and provide precise mechanical stress^17^.

These factors form the bottleneck in current abilities to research MIS phenomena and develop MIS protocols. Developing a robotic system capable of treating plants individually would potentially help overcome these challenges and lead to rapid development of MIS induction protocols.

This work outlines the development of several robotic platforms for stress induction. Using these platforms, a set of MIS protocols are developed, followed by evaluation protocols for plant morphology, metabolites, and element content. The results of evaluating these platforms and protocols on the use-case of basil plant production in situations resembling laboratory and commercial setups are reported. Finally, conclusions from our observations are drawn and presented in a detailed discussion, along with suggestions for future adjustments to MIS protocols and growing conditions.

### Related work

The literature exploring the effect of stress in general, and mechanical stress in particular, is vast. Several review papers, published from the 1980s onward, summarize the scientific efforts in this research area well^6,15,16^. Nevertheless, to the best of the authors’ knowledge, very little is available to be translated into applicable, automated MIS protocols, and the metabolic, nutrient, and sensory aspects of MIS in plants have been little explored.

Jaffe^18^ have explored in their work the aspects of prolongation of mechanical stimuli with *Phaseolus vulgaris* beans. They showed that six minutes of mechanical stimulation ceases stem elongation, and that it recovers after a period of no stimulation. Garner and Björkman^19^ have looked into the time intervals between mechanical treatments of tomato seedlings and found that intervals shorter than ten minutes are equivalent in effect to continuous treatment. Koch et al.^5^ have reported effects of mechanical stress induction in potted herbs, showing a significantly reduced elongation growth. Paul-Victor and Rowe^20^ have explored the effect of mechanical perturbation on the stem flexibility and diameter of *Arabidopsis thaliana* in potted growing conditions, and showed that mechanically perturbed plants grow to be ‘short and flexible’.

As for the chemical aspects of MIS, Seljåsen et al.^14^ found that mechanical stress in the post-harvest stage influences the levels of terpenes and sugars in carrot varieties, thereby inducing changes in their sensory profile. The results corresponded to the sensory results measured by an expert taste panel, with increased bitterness and earthiness reported in the product subjected to MIS.

To the best of the authors’ knowledge, precise automation of mechanical perturbation of plants is rather rare in the literature. Nevertheless, it does provide a few examples of such systems. Koch et al.^5^ have induced mechanical stress using light cloths connected to rails, continuously treating plants 108 times a day in commercial greenhouse conditions. Paul-Victor and Rowe^20^ introduced an automated system consisting of a chariot mounted on rails above the plant pots, with a single polythene sheet gently brushing the plants. Wang et al.^21^ performed a similar experiment on *Arabidopsis thaliana*, describing a similar system of automation. The laboratory experiments described^20,21^ have not focused on the ability to alter robotic motion design, and provided either a brief description of the automation or used a fixed robotic design, unable to be altered. Moreover, none of the publications mentioned have investigated the changes in chemical content of the plants undergoing treatment, but focused on the morphological aspects of the plant post-treatment.

Therefore, given the rarity of previous similar work to design mechanical platforms for precise MIS induction, this work refers to similar agricultural robotics applications where continuous physical manipulation of plants is required while they are growing. We use two common agricultural operations undergoing automation efforts in the agri-robotics literature: autonomous weeding robots and autonomous harvesting robots. These applications are chosen based on the relatively advanced, fully integrated robots available in the literature for these operations^22–26^. Weeding robots are often equipped with a two or three degrees-of-freedom (DoF) manipulator attached to a mobile platform to treat the weeds in the field from a top-down viewpoint^24–26^. The top-down viewpoint is ideal for both extraction of the weeds and increasing detection rates, due to minimization of the natural light abstractions. The top viewpoint is also beneficial for the current MIS application, as it is the tops of the plants that are to be manipulated. Harvesting robots in greenhouse crops are most often equipped with three to seven DoF robotic arms placed on a mobile platform navigating through the greenhouse^22,23,27^. The increased number of degrees of freedom are used for complex manipulations and target approach path planning^28^, which might be of benefit for exploring variations of possible MIS manipulation strategies. Both applications often use advanced perception sensors^29–32^, vision algorithms^33,34^, dynamic sensing strategies^35^ and control algorithms^27,28,36,37^ for navigation of the end-effector to the exact harvesting or weeding location, which is most often rapidly changing in the field. While manipulator control and path-planning algorithms were used in this work to navigate the end-effector to the required location for mechanical stress induction, the current platform does not require integration of expensive vision and perception sensors, due to the static location of the plants and their relatively low variability in plant height and locations. The outlined perception algorithms can be taken into consideration for more rapidly growing plants where the adjustment of manipulation height needs to be done in a dynamic manner. As outlined in “Materials and Methods” section, possible sensory infrastructure in the form of integrated cameras to accommodate these potential future needs were introduced in the development of the platform. Finally, for the design of the end-effector for mechanical stress induction, this study relies on the work of Ref.^5^, as also outlined in “Materials and methods” below.

### Contribution

**Figure 1:**
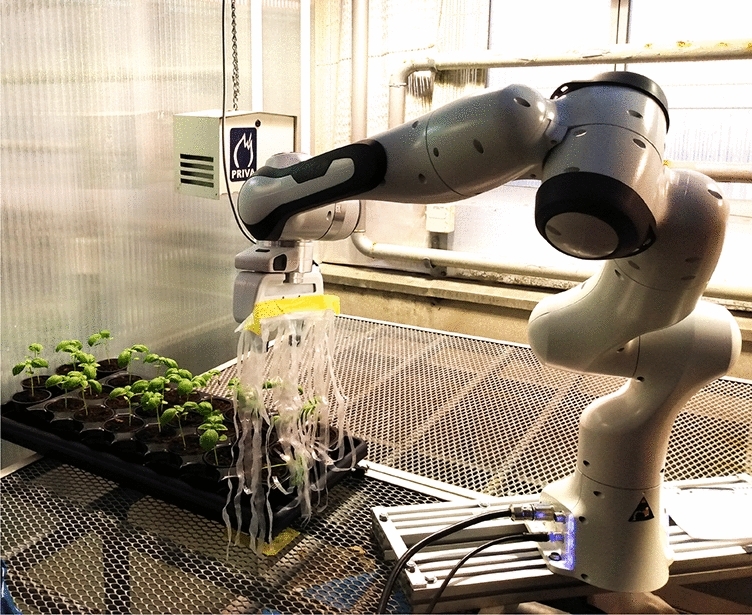
Seven-DoF robotic arm equipped with a stroking end-effector, placed in a commercial-type basil greenhouse setting.

**Figure 2:**
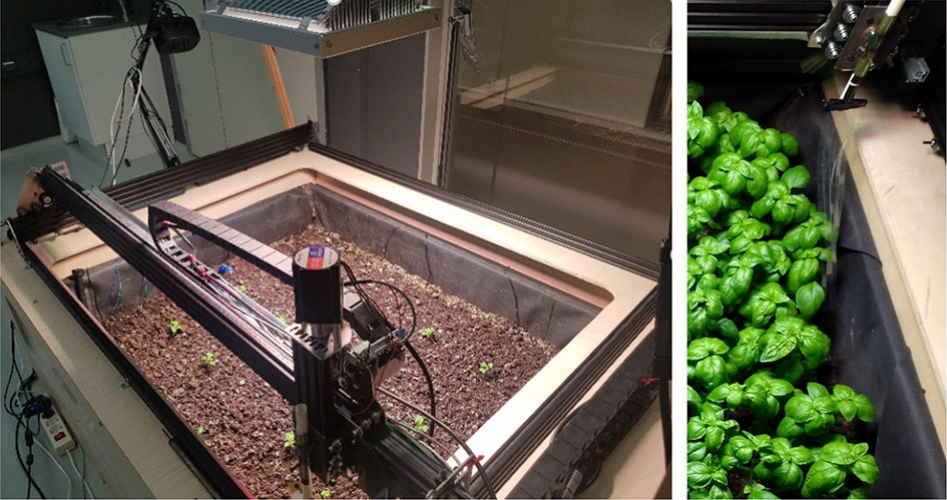
LOMAS++ cultivation testbed equipped with three-DoF robotic manipulator and a stroking plastic end-effector for mechanical stress induction. Left: the growing bed. Right: the stroking end-effector.

This paper’s contribution is threefold. First, it presents two types of robotic platforms (Figs. 1 and 2) for MIS research in controlled greenhouse and growing bed conditions. The platforms developed provide a high resolution of treatment, up to a single pot, allow variation in mechanical stress aspects such as speed, frequency, and torque, and can perform continuously up to a thousand motions a day. The platforms can potentially provide researchers with a tool for investigating MIS phenomena for rapid protocol development. The systems are developed for controlled (indoor) cultivation environments (potted or growing bed conditions), but can be adapted for outdoor stimulation as well. Commercial growers can benefit from adaptation of the indoor protocols to replicate the effect in their growing conditions.

Second, it presents an evaluation protocol and experimental design for investigating three aspects of plant growth: plant morphology, element content, and metabolite profile. The evaluation protocol looks into previously discussed variations in mechanical stress frequency and type, as well as new aspects such as prolongation and starting time. These aspects are analyzed throughout the growing period, with samples taken on multiple occasions along the growing cycle. The unique metabolite and element extraction protocols provide us with the ability to analyze the components affecting health, nutritional content, and taste of the product. The protocol, designed to suit small potted plants (such as herbaceous and seedlings), outlines the spatial positioning of the pots. The protocol is also adapted to growing bed conditions and outlines localization within the growing bed, as well as the placement of control groups. The number of plants used for analysis is discussed, along with the measurement collection and analysis protocols.

Finally, this paper provides the results of a use-case for the protocols outlined above, with basil plants grown in greenhouse and growing bed conditions. Chemical alterations in basil plants have a direct effect on sensory experience, the main reason basil is grown in commercial production for the food industry. Production of basil plants with shorter and lighter stems while retaining or increasing the weight of the leaves minimizes production waste and increases the valuable output. In this paper we show how the new protocols help achieve these favorable attributes. Changes in plant morphology and chemical content that have a direct link to plant health and nutritional content are presented.

## Materials and methods

### Autonomous robotics system for MIS

For repeated mechanical treatment to be commercially feasible it needs to be automated. But to receive similar treatment using brushing systems available today requires the tops of all brushed plants to be uniform. Growing different species and cultivars in the same place makes a similar treatment difficult using current systems^17^.

These factors greatly limit our ability to research the associated phenomena, and achieve robust and detailed protocols that will lead to favorable results. Introduction of a sophisticated robotic system providing individual treatment can potentially resolve this bottleneck and lead to rapid development of mechanical stress-induction protocols. Such protocols will allow researchers interested in the phenomena to have full control over the mechanical stimuli and experimental design size. Their further implementation on a simpler autonomous system providing fewer degrees of freedom will allow for introduction of the protocols into commercial practice.

As a result, this paper presents two autonomous platforms. The first is a robotic platform allowing seven-DoF motions, placed in a greenhouse setting. The second is a growing bed equipped with a three-DoF autonomous robotic arm, allowing the motions developed in the greenhouse to be reimplemented in a setting more closely resembling a commercial one, as outlined below.

#### Pot-based treatment: greenhouse conditions

A seven-DoF Franka Emika robotic arm, equipped with a stroking end-effector consisting of a row of plastic strings, inspired by the string design of Koch et al.^5^ (Fig. 1), was placed in an educational greenhouse setting resembling a commercial greenhouse. The greenhouse provided centralized control of growing lights, temperature, and humidity, and was equipped with metal halide grow-lights operating 12 h a day. The temperature in the growing cell was kept between 24 $^\circ$C (daytime) and 16 $^\circ$C (night time) during all experiments. Irrigation was performed using sub-irrigated trays.

All robotic arm motions were planned using the MoveIt motion-planning framework^38^, using the 3D visualization tool RVIZ for the Robotic Operating System (ROS)^39^. For robust operation, motion planning was performed in joint space using the RRTConnect planner^40^. For more details on the operational aspects of the platform, the reader is kindly asked to refer to the authors’ previous publication^41^.

#### Growing Bed Treatment

An autonomous cultivation bed (“LOMAS++”, Fig. 2)^42,43^, with growing area of approximately 1.2 m$^2$, capable of holding plants up to 30–35 cm tall, was placed in laboratory conditions. The cultivation bed was equipped with a growing light (model TELOS-0006A with light output PPF of 366 $\upmu$mol) and with different sensors (including gas, pH, ambient temperature, soil moisture, soil temperature, soil humidity, light, and quantum sensors) to maintain a controlled growing environment^44^. TELOS-0006A is a modern and efficient LED grow-light, suitable for top-down growing applications in commercial greenhouses and harsh environments. The average PPFD of 9.39 $\upmu$mol/m$^2$/s is obtained for a distance from the lamp of 4 m, with a coverage area (per unit) spanning 5.8 m $\times$ 5.8 m. The testbed is also equipped with a custom built-in four-channel spectral imaging setup, based on a Raspberry Pi and used for phenotyping tasks^45^, mounted over the manual manipulator arm (Fig. 2). This can be automated using a PID control with Type-2 fuzzy logic (T2-F-PID) for vibration control^46,47^. The growing bed was coupled with a fully automated three-DoF robotic manipulator (Fig. 2) equipped with a stroking end-effector, similar in material and shape to the one used in the greenhouse setup, to induce mechanical stress in the plants. The robotic arm is driven by stepper motors and actuators, a micro-controller, and a Raspberry Pi computer, while the low-level control and logic is based on ROS^39^. The telemetry sensors are interfaced through the Firmata protocol^48^, and the machine through the G-protocol. The system functions with X, Y, and Z-direction movements along the tracks, gantry, and cross-slides (hence acquiring three DoF). The arm control is performed in Cartesian coordinates.

### Plant quality measures

The following aspects of plant response to MIS were explored: (1) changes in plant morphology, (2) changes in metabolites, and (3) changes in major and trace elements. The methods for measuring these changes are outlined below.

#### Morphological measures

Changes in plant morphology were measured according to the protocol outlined in Kurtser et al.,^41^. Two types of measures were collected: non-destructive measures, and destructive measures. The non-destructive measures, which can be measured several times during the growing cycle with no physical harm to the plants, include (a) inter-node length, (b) stem length, and (c) inter-node diameter. Upon plant harvest the following measures are taken: (a) plant and leaf fresh weight, and (b) dry weight. For more details on the specific measurement protocol please refer to our previous publication^41^.

#### Metabolite profiling

The analysis of plant metabolism was done as described previously^4^, and consisted of five steps: (1) extraction of polar metabolites and two-step derivatization, (2) gas-chromatography ultra-high-resolution/accurate mass spectrometry (GC-HRAM) analysis, (3) GC-HRAM data pre-processing, (4) GC-HRAM performance checking, and (5) metabolite identification and generation of the resulting dataset.

Extraction of polar metabolites and two-step derivatization: samples (25 ± 4 mg) were added with cold methanol containing valine-d8 (internal standard), and extraction was carried out in an ultrasonic bath for 10 min. After centrifugation (11,000*g*, 10 min), supernatants (400 $\upmu$L) were collected and mixed with chloroform (220 $\upmu$L, $-20$ $^{\circ }$C) and water (440 $\upmu$L, 4 $^{\circ }$C). Samples were vortexed, and after centrifugation (2200*g*, 15 min), 150 $\upmu$L of the upper phase (aqueous phase) were transferred to GC vials. Pooled extracts to be used as quality controls (QC) were also prepared by mixing equal aliquots from the upper phase of the samples. Extracts were further dried under a nitrogen stream and then converted into methoxime (MEOX) and trimethylsilyl (TMS) derivatives. Oximation was performed with 25 $\upmu$L of methoxyamine hydrochloride (20 mg/mL in pyridine, 45 $^{\circ }$C, 1 h) and silylation with 25 $\upmu$L of *N*-Methyl-*N*-(trimethylsilyl)trifluoroacetamide (45 $^{\circ }$C, 1 h). After derivatization, a mixture containing odd n-alkanes (C11 to C25, 10 $\upmu$L/mL in hexane) for further calculation of retention index.

Gas-chromatography ultra-high-resolution/accurate mass spectrometry (GC-HRAM) analysis: derivatized samples were analyzed by GC-HRAM using a Q Exactive GC Orbitrap system (Thermo Scientific) equipped with an Rxi Guard column (10 m $\times$ 0.37 mm, 0.25 mm i.d., Restek) and a HP-5MS capillary column (30 m $\times$ 0.25 mm, 0.25 $\upmu$m i.d., Agilent Technologies). Injection (1 $\upmu$L; TriPlus RSH Autosampler, Thermo) was performed in split mode (1:20). The oven temperature was held at 70 $^{\circ }$C for 5 min and increased to 260 $^{\circ }$C at 10 $^{\circ }$C/min, then to 300 $^{\circ }$C at 40 $^{\circ }$C/min, and then was held for 5 min. Helium was used as a carrier gas (2.0 mL/min). The MS detector was operated in EI positive mode, with a scan range of 50–500 *m/z* and resolution of 60,000. The transfer line and ion source were maintained at 280 $^{\circ }$C.

GC-HRAM data pre-processing: pre-processing steps were performed using the ADAP-GC 4.0 module within the framework of MZmine 2.51. The pre-processing workflow included raw file import (mzML data) followed by mass detection, construction of extracted ion chromatograms, peak detection, deconvolution, alignment, filtering, and feature identification. The following parameters were applied: (1) mass detection of centroid data; (2) chromatogram building with a minimum group size of five scans, group intensity threshold and minimum highest intensity at 5000, and *m/z* tolerance set at 0.001; (3) chromatogram deconvolution using the wavelets (ADAP) method, with a signal/noise ratio of five, minimum feature height of 10,000, coefficient/area threshold of 100, peak duration range between 0.1 and 0.3 min, and retention time (RT) wavelet range up to 0.03 min; (4) spectral deconvolution using a hierarchical clustering method with a cluster distance of 0.01 min, minimum intensity of 5000, and shape similarity tolerance of 50; (5) peak alignment using the Join aligner algorithm module with RT tolerance of 0.002 min (weight 2) and *m/z* tolerance of 0.15 (weight 1); (6) filtering to maintain only the features that appeared in 75% of samples; and (7) identification of metabolites using a custom database search with a *m/z* tolerance of 0.002 (base peak) and RT tolerance of 0.2 min.

The peak list was then exported (RT, *m/z* of base peak and peak area) for further processing steps, which included calculation of retention index and normalization by sample weight. Normalization using the relative peak area of the *m/z*-RT pair 152.1704-10.2 (relative to IS) was also performed. Features without the characteristic m/z fragment at 73.046 (TMS moiety) and those found at high levels on blank or with RSD > 30% in the pooled QC samples were also excluded from the peak list.

GC-HRAM performance check: QC samples were injected after every tenth sample. Process blank (extraction blank) and system suitability blank (hexane) were analyzed at the beginning and end of the analysis batch. The relative standard deviation (RSD) of the IS on QC samples and extraction blanks were 3.9% and 8.4% on the cultivation bed and greenhouse conditions, respectively.

Metabolite identification and resulting dataset: After data pre-processing and normalization by weight and IS, a .csv file was generated separately for Experiment 2 (growing bed) and Experiment 3 (greenhouse), containing the samples in rows and the relative levels of each detected feature/metabolite in columns. Metabolites of interest had their identity explored by comparing the full spectra and retention time of peaks with those obtained for the in-house analytical standards. Two additional levels of annotation (levels 2 and 3) were defined for the non-identified features: level 2 included features putatively identified by matching their spectra and RI with those available in the Golm Metabolome Database (GMD) using recommended parameters for mass spectral matching—except for substructure prediction and $\Delta RI$, which were increased to 90% and 20, respectively; level 3 was defined for the remaining non-identified compounds which were classified according to functional group predictions (> 90% probability), by searching for specific m/z fragments described using the trained decision trees available on GMD.

#### Major and trace element analysis

Approximately 0.5 g of dried leaves from each replicate were homogenized and transferred to Teflon beakers. To each sample, 6 mL of concentrated HCl and 2 mL of concentrated HNO$_3$ were then added, and the mixture was heated to about 60 $^\circ$C. After 2 h, another 6 mL concentrated HCl and 2 mL concentrated HNO$_3$ were added, and the mixture was heated for another 2 h. Then the digested samples were transferred to 50 mL polypropylene test tubes (Sarstedt). The Teflon beakers were rinsed with deionized water (18.2 M $\Omega$) that was then collected, and finally the volume of each sample was adjusted to 50 mL.

After settling for 24 h, 1 mL was drawn and analyzed for Li, Be, Na, Mg, K, Ca, Rb, Sr, Ba, Al, V, Cr, Mn, Fe, Co, Ni, Cu, Zn, Ge, As, Se, Mo, Ag, Cd, Te, Tl, Pb, Bi, and U, after appropriate dilution using 1% concentrated nitric acid in deionized water (18.2 M$\Omega$). Analysis was performed using ICP-MS (Agilent 7500 cx). Dilute solutions of the Merck 10580 multi-element standard VI were used for external calibration. Calibration was performed in the interval 1–10,000 ppb for Ca, 0.1–1000 for Be, Fe, Zn, As, Se, 0.01–1000 for Na, K, Mg, Al, Mn, and 0.01–100 for the remaining elements. Elements prone to suffer from di- and polyatomic interferences, denoted with [2] in the results, were analyzed in collision mode using helium as the collision gas at a fixed flow rate of 5 mL/min. Rhodium was used as the internal standard for all elements and was added to samples and standard solutions prior to analysis.

## Experiment design

**Figure 3:**
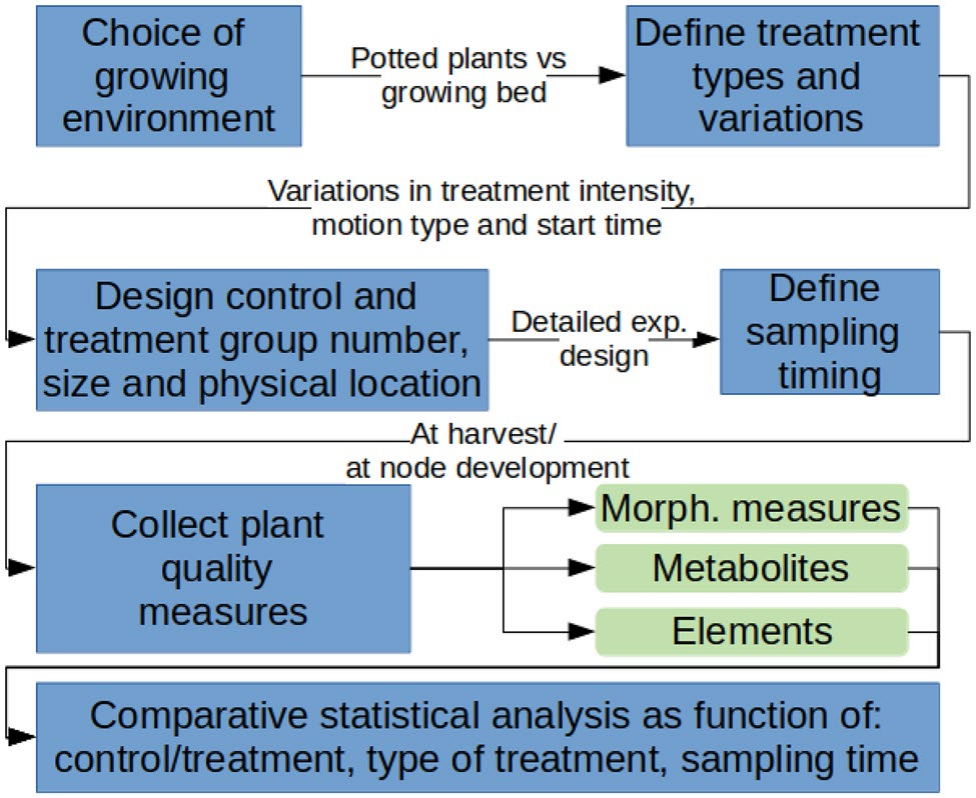
Overall evaluation protocol for mechanical stress using the robotic systems developed, and the illustrative experiment design outlined in Experiments 1–3.

Three experiments were conducted with the aim of exploring different variations of mechanical stress induction. The experimental designs followed the overall evaluation protocol described in Fig. 3. The experiments were designed to both evaluate how variations in mechanical stress affect plant growth and to repeat and verify previously obtained results. All experiments were performed on basil plants, and adhered to relevant plant ethics guidelines. Basil is a popular herb used mostly for its leaves. Therefore, production of basil plants with large leaves and favorable chemical content is of high importance, partially due to the taste and aroma implications—the main usage of basil in domestic and commercial cooking. These experiments show how some of these favorable attributes can be achieved using the platforms developed.

### Experiment 1: MIS motion variations in potted plant conditions

Experiment 1 was conducted in the greenhouse using a seven-DoF robotic manipulator, as described in “Methods and Materials” Section (“Pot-based treatment: greenhouse conditions”). The goal of the experiment is to explore how differences in the frequency of mechanical motions and their type affect the morphology and element content aspects of plant growth. The experiment and morphological results were previously published in Kurtser et al.^41^, and therefore only the necessary details required for a comparative evaluation of the results are presented here. In the subsection “Effect of stroking on element absorption”, the outcomes of the element analysis that were not published previously, are also reported.

**Figure 4:**
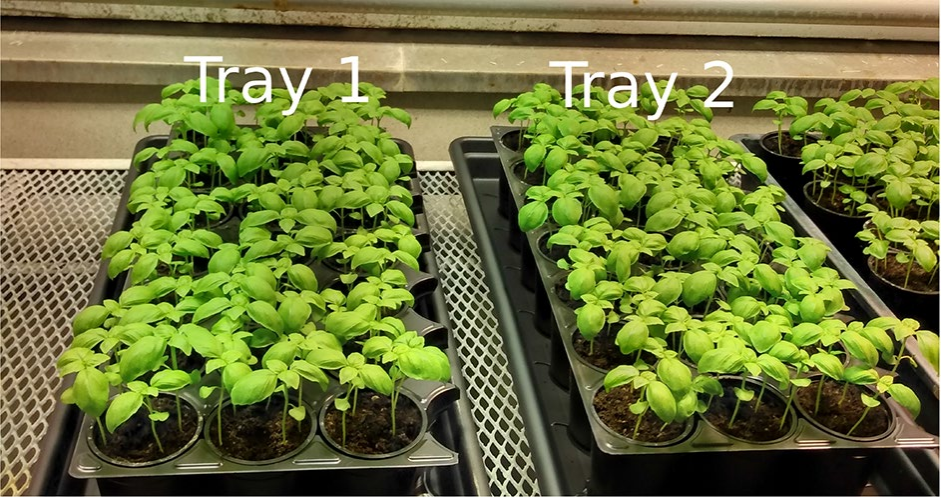
Treatment groups and trays prior to treatment starting. The first five rows of *Tray1* and *Tray2* are the 100 t/day and 2 t/day groups, respectively. The rest of the plants in the trays are Control1 and Control2 groups, respectively.

**Figure 5:**
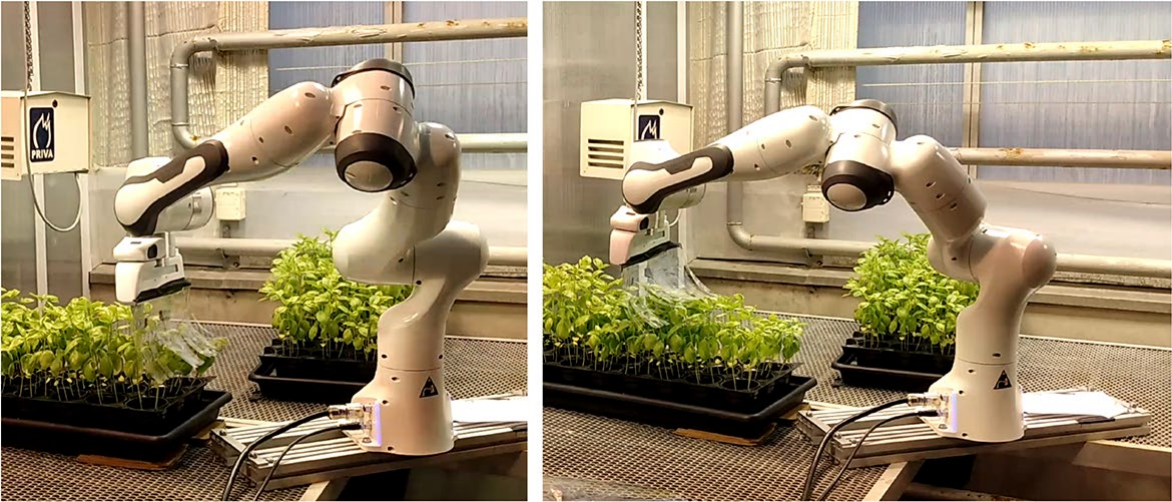
Two types of *“robot stroking”* motions: dipping (left) and plant-top stroking (right).

A total of 50 pots (7 cm diameter) were filled with limed and fertilized peat soil, and sown with 5–10 seeds of basil per pot, variety Genovese. The pots were thinned after 11 days down to 3–5 seedlings. 30 days after sowing, the pots were randomly assigned into six groups according to Table 1, including four treatment groups and two control groups. The experiment includes variation in physical location of the pots (two trays; Fig. 4) motion types (stroking and dipping; Fig. 5) and motion frequency (repetitions 100 times a day or two times a day).

**Table 1:** Description of four treatment groups (T) and two control groups (C) for Experiment 1.

Group	Tray	Rows	Motion type	Motion frequency	No. of pots
T1—stroking	1	2, 3	Stroking	100 t/day	6
T1—dipping	1	1, 4, 5	Dipping	100 t/day	9
C1	1	6, 7, 8	None	0 t/day	10$^{\text {a}}$
T2—stroking	2	2, 3	Stroking	2 t/day	6
T2—dipping	2	1, 4, 5	Dipping	2 t/day	9
C2	2	6, 7, 8	None	0 t/day	10$^{\text {a}}$

$^{\text {a}}$Row 8 consisted of four pots, while rows 1–7 consisted of three pots each.

Treatment began 31 days after sowing and lasted a total of 27 days. Non-destructive morphological measures were collected, according to the methods described in “Morphological measures”, on the last day of treatment (58 days after sowing), while destructive morphological measures were collected upon harvest on the following day.

Upon collection of dry weight measurements, eight samples were collected for element analysis according to the methods outlined above “Major and trace element analysis”.

### Experiment 2: MIS motion prolongation in growing bed conditions

Experiment 2 was conducted in the test-bed setting using a three-DoF robotic manipulator as described above “Growing bed treatment”. The goal of the experiment was to explore the optimal time in the plant growth cycle to start treatment.

**Figure 6:**
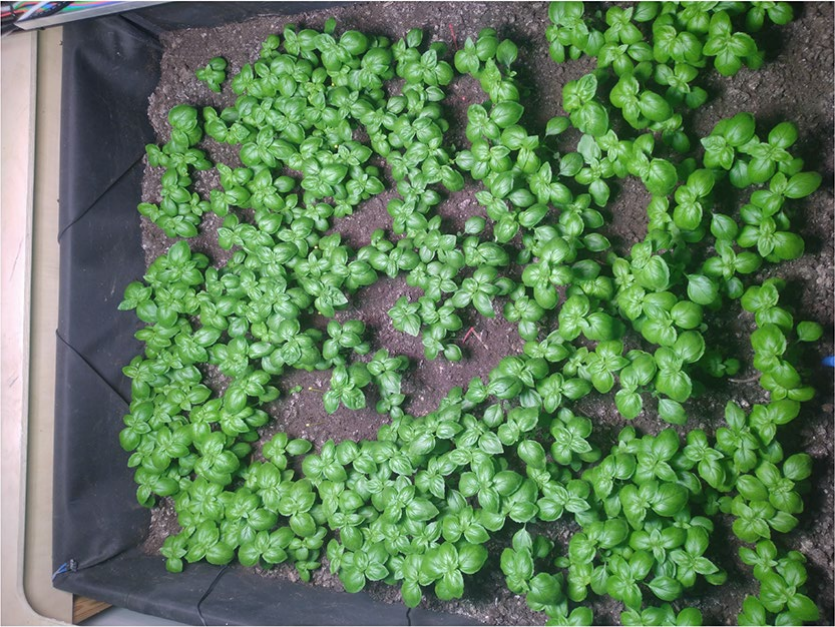
Lomas growing bed as observed in the fourth week from the start of the experiment.

**Figure 7:**
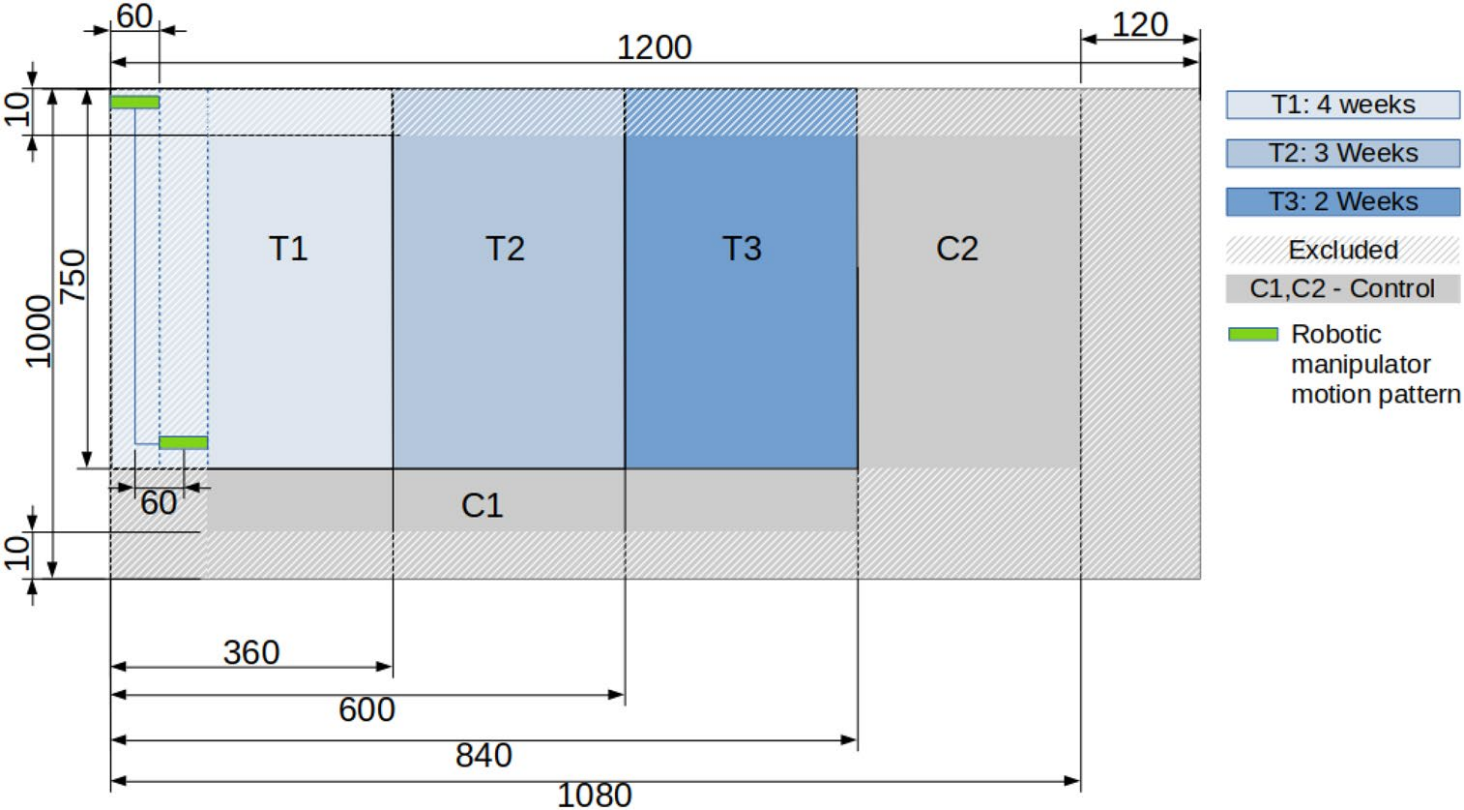
Description of three treatment groups (T) and two control groups (C) of Experiment 2.

The growing bed (Fig. 6), sized 1080 mm $\times$ 1000 mm, was sown with basil seeds in a random fashion. From the point a sufficient number of plants had grown their first pair of leaves, the bed was pruned repeatedly to eliminate the growth of new plants and ensure homogeneous growing. A total of around 200 plants remained in the bed, and were split into five groups (three treatment groups—T1, T2, and T3—and two controls—C1 and C2) according to their spatial locations, as described in Fig. 7. Plants growing up to 10–12 cm distance from the perimeter of the growing bed were excluded from the experiment to exclude any inhomogeneity aspects resulting from the presence of the growing bed walls and soil sensors. Treatment began from the point of full development of the first pair of leaves, at a rate of 100 times a day, during the daytime hours of approximately 9:00–16:00 and only on weekdays due to safety considerations in laboratory conditions where untrained staff and students are present.

Group T1 received four weeks of mechanical treatment, while group T2 started receiving treatment a week after group T1, and received a total of 3 weeks of treatment. Group T3 started receiving treatment a week after group T2 (2 weeks after group T1) and received a total of 2 weeks of treatment. Control groups C1 and C2 were spatially distributed along the growing bed to eliminate the possible affect of inhomogeneity inside the growing bed.

At the end of treatment, ten random plants were chosen from each group for data collection. Non destructive morphological measures were acquired according to the methods described in the “Morphological measures” Section. For metabolomic analysis, the youngest fully developed leaf of randomly selected plants was taken at each sampling point. The leaf was immediately frozen in liquid nitrogen and stored at $-80$ $^\circ$C for further metabolite extraction according to the protocol described in “Metabolite profiling” Section. Dry samples of leaves were then analyzed for elements according to the protocol described in “Major and trace element analysis” Section.

### Experiment 3: MIS growth modeling in potted plants conditions

Experiment 3 was conducted in the greenhouse in conditions resembling Experiment 1, with the following alterations. First, the stroking material was substituted with a softer material. Second, the plants were pruned down to a single plant per pot (versus 3–5 in Experiment 1). Third, no alterations in treatment frequency were introduced between the trays, and both were treated 100 times a day. The treatment groups were also altered as described in Table 2. The goal of the experiment was to explore how the plant measures change during the growing cycle as indicated by non-destructive morphological measures. Therefore, two sampling points were performed along the growing cycle upon development of a new pair of leaves and an additional sampling point was performed at the end of the experiment. Youngest leaf sampling was performed as described in Experiment 2. Since the sampling for metabolite analysis require physical removal of parts of a leaf, the sampling itself is considered as a possible additional plant stress that could mask the results of the mechanical stress induction, as the removal of leaves might influence the plant’s metabolite profile, and general growth. Therefore, the plants were separated into two types of sampling groups: (1) those plants that were sampled on all three sampling occasions (i.e., leaves were removed three times from the same plants) and (2) those plants that were sampled only once (either on the first, second or final sampling point). The sampling strategy is highlighted in Table 2.

**Table 2:** Experiment 3 treatment group distribution.

Motion type	Leaf samples	Tray	No. of pots
Dipping	1	1	3
		2	3
	2	1	2
		2	3
	3	1	4
		2	3
Stroking	1	1	2
		2	2
	2	1	3
		2	2
	3	1	1
		2	2
Control (None)	1	1	5
		2	5
	2	1	3
		2	3
	3	1	3
		2	3

* Row 8 consisted of five pots, while rows 1–7 consisted of three pots each.

## Results

### Morphological alterations

**Figure 8:**
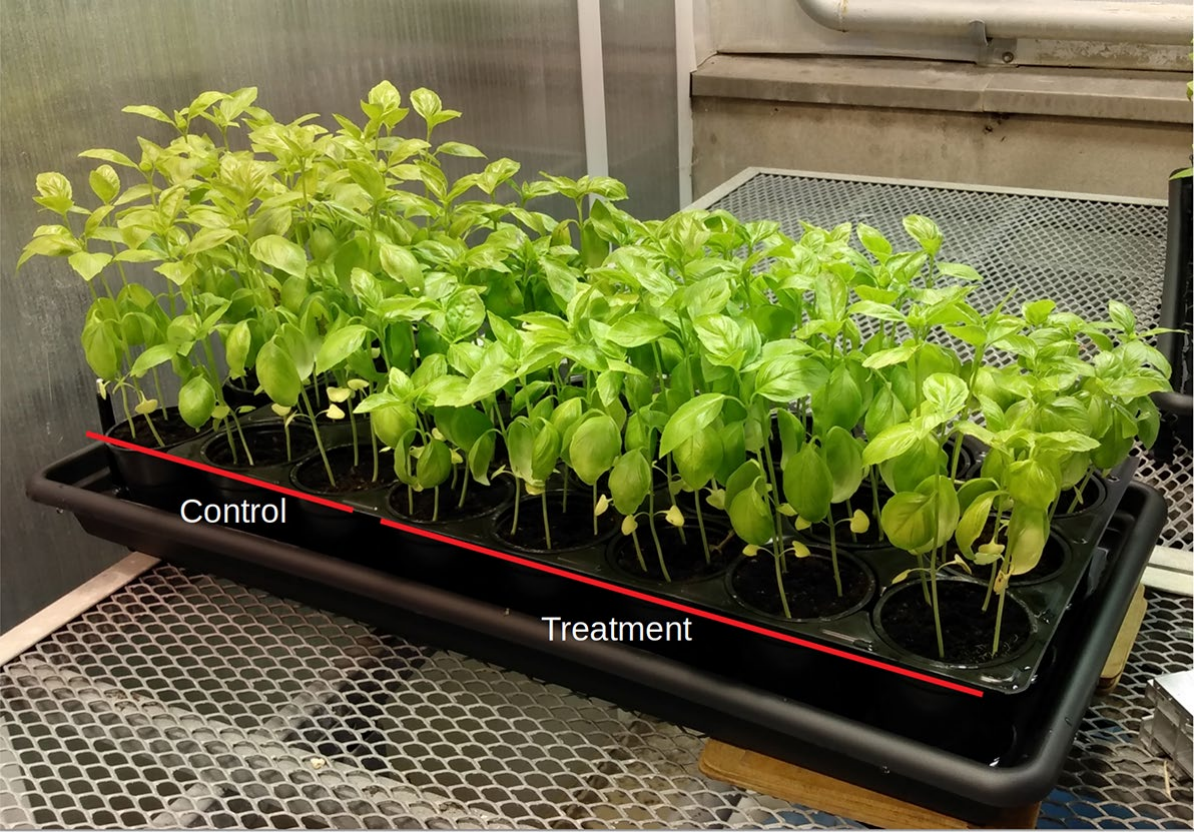
Visual evaluation of *Tray1* on the day of measurement, 58 days after sowing, four weeks after the start of treatment.

Visual evaluation of the trays at the end of Experiment 1 (Fig. 8) reveals clear differences in plant height between the “stroked” plants, compared to the control. Similar results were obtained for Experiment 3. Below the morphological results are outlined for each of the protocol variables chosen in Experiments 1–3.

#### Control group comparison

For Experiment 1 and 3, results show no statistically significant differences in any of the morphological measures between the control groups, suggesting the conditions in which plants were placed in *Tray1* and *Tray2* are similar.

For Experiment 2, performed in the growing bed, no statistically significant differences in most morphological measures were found between the control groups. The exceptions are the measures of the fourth internode, and overall stem length that are borderline significant ($p=0.0254$, and $p = 0.0068$, accordingly).

#### Stroking frequency and motion type variations

Variations in stroking frequency, performed in Experiment 1, showed no statistically significant differences between the group treated 2 t/day and its corresponding control group. However, for the 100 t/day treatment group, inter-node distances were found to be 31% shorter and overall stem length 50% shorter, compared to the corresponding control group. These results suggest that a 2 t/day treatment is not sufficient to produce significant changes in plant morphology, while 100 t/day results in significant differences in stem length. Stem diameter did not show any significant changes at any treatment frequency. These results support the findings by Victor and Rowe^20^, which showed stem diameter not to be affected by mechanical stress. When the fresh weight of all plants in a single pot were summed, a reduction of 8% (statistically significant) in overall plant weight was found for the pots treated 100 t/day. The difference in fresh leaf weight remained statistically insignificant. These results suggest a favorable reduction in plant weight while retaining leaf weight, and therefore minimizing production waste.

The results of variations in stroking motion types showed an increased morphological reaction to the dipping motions. Dipping motions performed frequently (100 t/day) particularly showed a significant shortening in stem length, of 42% compared to the corresponding control group. Shortening of the first two inter-nodes by 8–13% for the dipping motion were found even for the lower treatment frequency of two times a day, a result that was not found to be significant for the stroking motion.

For more detailed morphological results of Experiment 1 the reader is encouraged to refer to the authors’ previous publication^41^.

#### Effects of motion start time and prolongation

**Figure 9:**
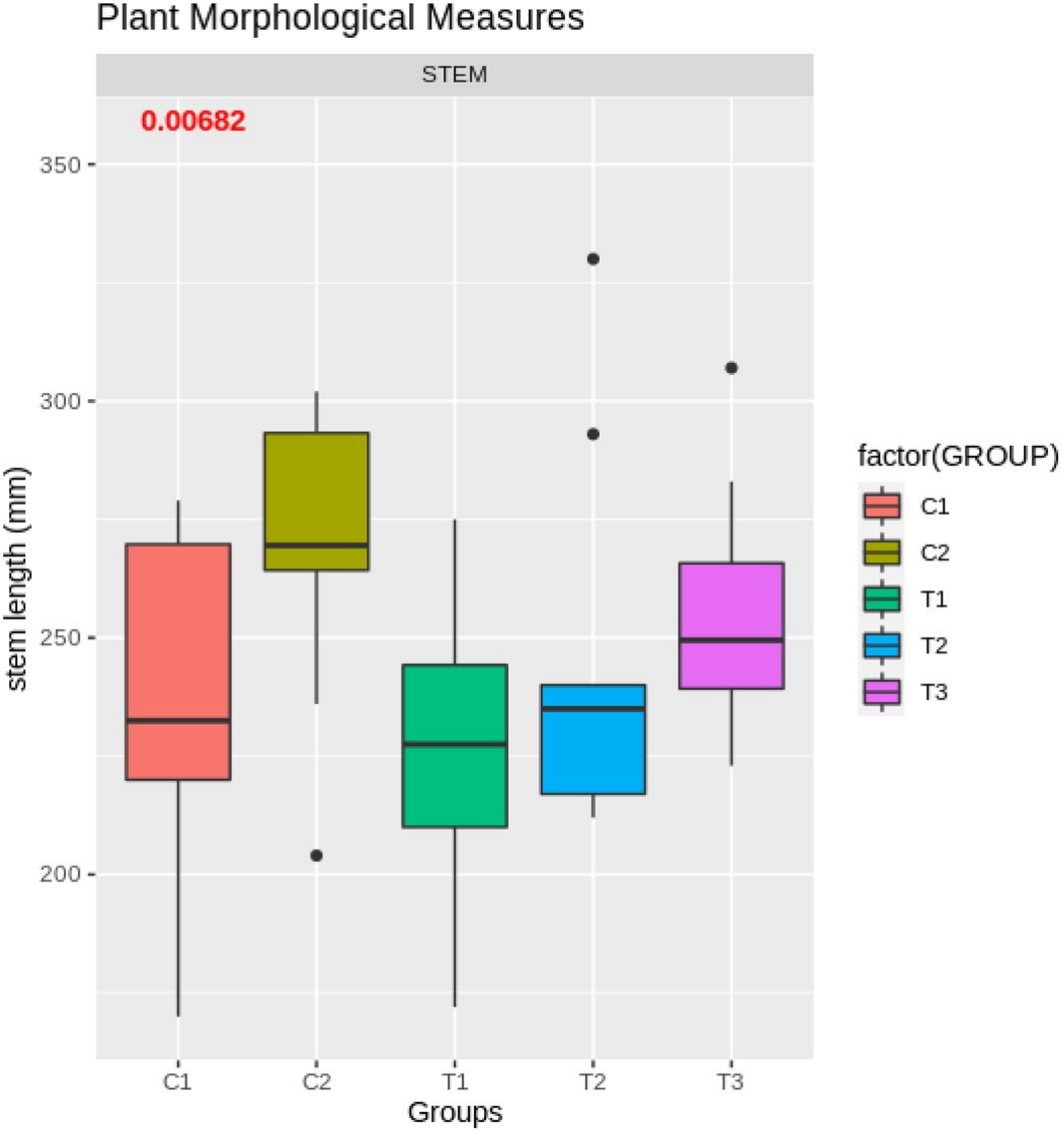
Test bed distribution of stem length (mm) as function of the treatment group (T1–T3) or control group (C1, C2).

In Experiment 2, performed in the growing bed, similar statistically significant differences were found in stem length (one-way ANOVA, $p<0.01$; Fig. 9). Group T1, receiving four weeks of treatment and starting first (see “Experiment 2: MIS motion prolongation in growing bed conditions”), also had statistically significantly shorter stems (on average by 8%; pairwise adjusted *t*-test, $p<0.05$) than group T3, which received only two weeks of treatment two weeks later in development. The differences between treatment groups and control groups were not statistically significant, suggesting that too small a sample size was analyzed. The borderline statistically significant difference between the control groups, and as a result the statistically significant difference between treatment group T1 and control group C2, suggest the possibilities of other intervening factors in the growing bed that might have influenced the results. These factors are outlined and elaborated on in the “Discussion” Section of this paper.

#### Growth modeling effects

In Experiment 3, the collection of morphological measures was performed twice during the growing cycle, at the point of emergence of the first and second pair of true leaves. Results show not only that the overall stem length (collected at the end of the experiment) is different between the treated groups and the control groups (average 184.3 mm vs. 244.5 mm, respectively; *t*-test, $p = 5$E$-9$), but the increase between the first and second measurements is significant as well (83 mm vs. 127.8 mm, *t*-test, $p = 1.2$E$-7$). This is further supported by the fact that the mechanical stress that began after the development of the first pair of leaves did not affect the first inter-node distance, which increased on average by 6–7 mm for both the control and the treatment groups (*t*-test, $p>0.1$). The second inter-node increased significantly less for the treated group, compared to the untreated (33 mm vs. 43 mm, *t*-test, $p = 5$E$-3$). The third inter-node was not fully developed at the time of the first measurements, but was found to be significantly longer for the control group compared to the treated group (55 mm vs. 31 mm, $p = 2$E$-6$) at the second measurement. These results suggest that MIS does not affect the already developed inter-node length, and increasingly effects not only the nodes developing subsequently, but also their speed of development.

### Metabolite analysis

#### Experiment 2: MIS motion prolongation in growing bed conditions

The entire metabolite dataset from Experiment 2 was comprised of a matrix containing the relative levels of 89 metabolites or features in 31 samples from five groups (C1, C2, T1, T2, and T3). Before statistical analysis, the missing values in the matrix (43, 1.6%) were replaced by 1/5 of the minimal values of their corresponding variables. Data was then log-transformed, and the resulting $89 \times 31$ matrix was explored to assess potential differences in the metabolite profile between groups. Firstly, the control groups (C1 and C2) were tested for significant differences in their metabolite levels. Since no significant differences were found between the levels of the 89 metabolites in C1 and C2 (*t*-test, $p < 0.05$), further analysis considered the two control groups as a single group.

**Figure 10:**
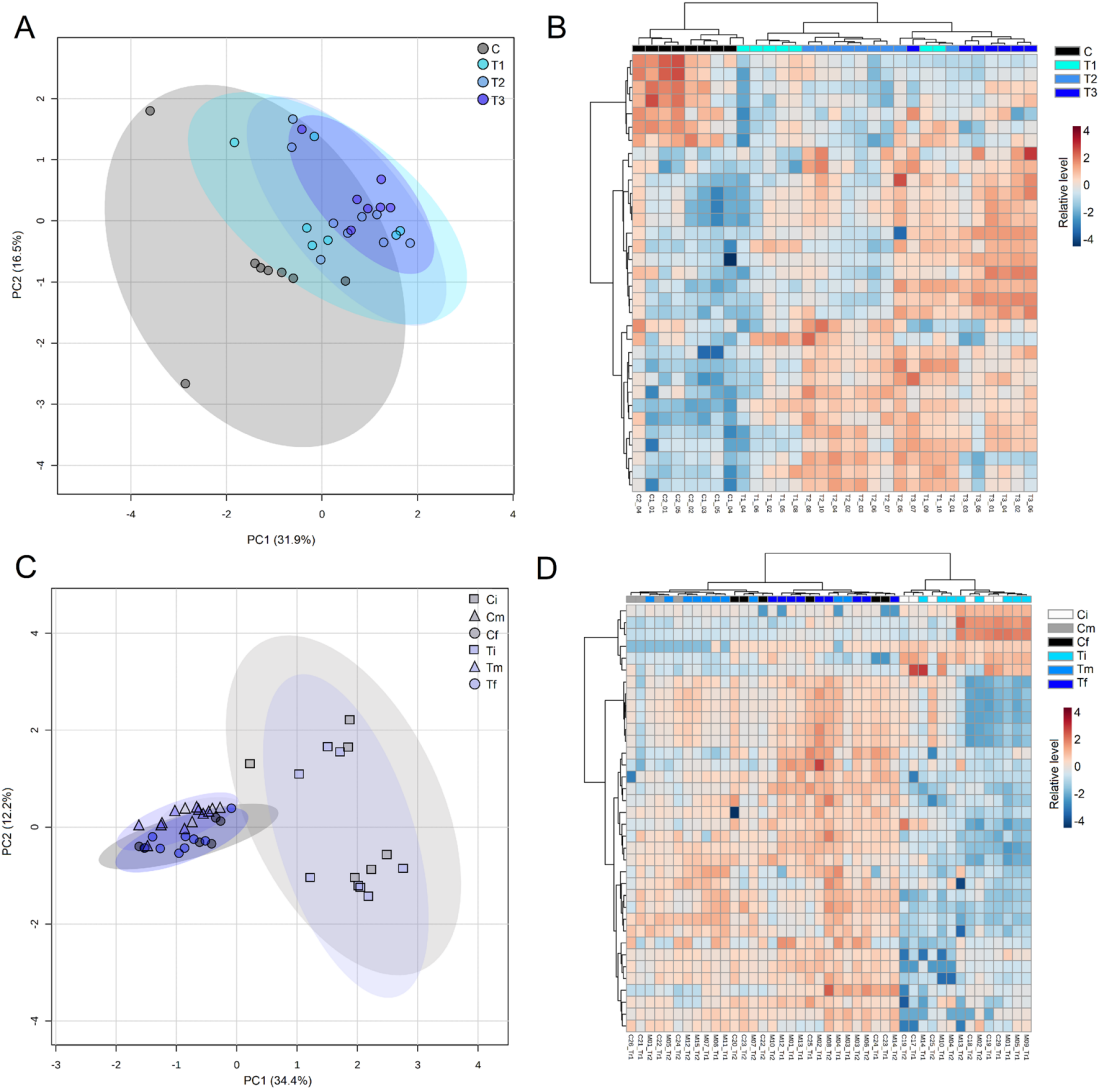
Metabolite profile of basil in Experiments 2 and 3. PCA scores plot of the entire metabolite dataset from (**A**) Experiment 2 and (**C**) Experiment 3. Heatmap with significant metabolites (ANOVA, FDR-adjusted $p < 0.05$) of (**B**) Experiment 2 (*n* = 33) and (**D**) Experiment 3 (*n* = 34). C: Control, T1: 4 weeks’ treatment, T2: 3 weeks’ treatment, T3: 2 weeks’ treatment (Experiment 2). Control and treated samples obtained at the first/initial (Ci, Ti), second/intermediary (Cm, Tm), and third/final (Cf, Tf) sampling point (Experiment 3). Figure generated using the open source program *Metaboanalyst*^50^.

Principal component analysis (PCA) was then applied to explore overall response patterns. The first two principal components explained 48.4% of the total variance among groups (Fig. 10A). Control groups were separated from treatment groups mainly along PC1, responsible for 31.9% of the total variance explained. Although there was no clear separation between T1, T2, and T3 in the two-dimensional space, the 95% confidence interval displayed in the PCA showed samples from T1 closer to control samples, followed by samples from T2 and T3. The biplot of the PCA model suggests that most of the metabolites were positively correlated with samples from treated groups, thereby suggesting higher accumulation of metabolites in treated samples compared to control samples (Supplementary Fig. S1A). The significant differences between the levels of metabolites among the groups was further explored. As shown in Table 3, 33 metabolites showed significant differences (ANOVA, FDR-adjusted $p < 0.05$). Confirming the study’s hypothesis, most of these significant metabolites had higher average values in the treated samples, as can be clearly seen in the heatmap (Fig. 10B).

Amino acids, including methionine, alanine, proline, serine, and tyrosine, showed higher levels in treated samples. The same pattern was observed for some organic acids, including malonic acid, malic acid, and tartaric acid. Treatment also induced accumulation of GABA, shikimic acid, and dehydroascorbic acid (DHA). On the other hand, control samples were characterized by increased levels of specific organic acids (e.g., pipecolic acid and glyceric acid), as well as 2-oxoglutarate (2-OG). The profile of sugars also differed among control and treated samples. Treatment induced the accumulation of sucrose and monosaccharides, such as glucose and galactose, while decreasing the levels of disaccharides including maltose (glucose + glucose) and lactose (glucose + galactose), compared to controls.

**Table 3:** Metabolites with significant differences (ANOVA, FDR-adjusted $p<0.05$) on Experiment 2 (LOMAS). Relative levels in relation to control samples.

Metabolite	ID level	q-value	Control	T1	T2	T3
2-Oxoglutarate (2-OG)	2	1.5E−04	1.0 ± 0.3	0.4 ± 0.1	0.3 ± 0.1	0.5 ± 0.2
5-Oxoproline	2	8.2E−04	1.0 ± 0.5	1.8 ± 0.7	2.5 ± 0.7	2.3 ± 0.9
Alanine	1	1.8E−04	1.0 ± 0.4	1.3 ± 0.6	2.6 ± 0.6	2.5 ± 0.6
beta-Alanine	1	9.6E−05	1.0 ± 0.3	1.7 ± 0.9	3.1 ± 0.7	1.0 ± 0.7
Dehydroascorbic acid (DHA)	2	9.9E−04	1.0 ± 0.2	1.3 ± 0.2	1.5 ± 0.2	1.6 ± 0.3
GABA	1	8.2E−04	1.0 ± 0.3	2.3 ± 0.9	2.5 ± 0.4	1.6 ± 0.5
Galactose	1	1.9E−03	1.0 ± 0.6	2.3 ± 1.1	1.8 ± 0.9	8.2 ± 2.4
Glucose	1	1.5E−02	1.0 ± 0.2	1.3 ± 0.2	1.3 ± 0.3	0.9 ± 0.1
Glyceric acid	2	1.5E−02	1.0 ± 0.2	0.8 ± 0.2	0.7 ± 0.1	0.7 ± 0.1
Glycerol	2	1.2E−04	1.0 ± 0.2	1.4 ± 0.1	1.5 ± 0.2	1.5 ± 0.2
Lactose (Glc + Gal)	1	8.5E−03	1.0 ± 0.4	0.6 ± 0.2	0.6 ± 0.1	0.5 ± 0.1
Malic acid	1	1.4E−02	1.0 ± 0.1	0.8 ± 0.1	1.1 ± 0.1	0.9 ± 0.1
Malonic acid	2	3.1E−03	1.0 ± 0.1	1.0 ± 0.1	1.3 ± 0.3	1.6 ± 0.4
Maltose (Glc + Glc)	2	8.0E−05	1.0 ± 0.4	0.4 ± 0.1	0.3 ± 0.1	0.3 ± 0.0
Methionine	2	2.7E−02	1.0 ± 0.1	1.0 ± 0.1	1.2 ± 0.2	1.2 ± 0.1
Myo-Inositol	2	4.4E−05	1.0 ± 0.1	1.7 ± 0.7	2.2 ± 0.4	1.7 ± 0.2
NI alcohol	3	6.6E−03	1.0 ± 0.3	1.2 ± 0.3	1.4 ± 0.2	1.7 ± 0.3
NI carboxylic acid	3	2.3E−02	1.0 ± 0.3	0.5 ± 0.2	0.5 ± 0.1	0.7 ± 0.4
NI sugar	3	8.2E−04	1.0 ± 0.1	1.1 ± 0.2	1.2 ± 0.1	1.4 ± 0.1
NI sugar	3	8.9E−03	1.0 ± 0.3	1.2 ± 0.3	1.4 ± 0.2	1.5 ± 0.2
NI sugar	3	5.4E−04	1.0 ± 0.4	1.6 ± 0.7	1.1 ± 0.6	4.1 ± 0.8
NI sugar	3	4.4E−05	1.0 ± 0.4	1.6 ± 0.7	1.4 ± 0.6	4.1 ± 0.9
NI sugar	3	4.7E−02	1.0 ± 0.3	0.7 ± 0.3	0.6 ± 0.2	0.6 ± 0.1
NI sugar	3	8.2E−04	1.0 ± 0.2	1.2 ± 0.2	1.5 ± 0.1	1.2 ± 0.1
NI sugar	3	2.1E−02	1.0 ± 0.2	1.2 ± 0.3	1.4 ± 0.4	1.5 ± 0.3
Pipecolic acid	2	1.5E−02	1.0 ± 0.5	0.5 ± 0.1	0.5 ± 0.1	0.6 ± 0.2
Proline	1	8.2E−04	1.0 ± 0.5	2.9 ± 2.4	6.0 ± 2.7	2.1 ± 1.2
Pyruvate	2	3.7E−03	1.0 ± 0.3	1.0 ± 0.2	0.9 ± 0.2	1.6 ± 0.3
Serine	1	8.9E−03	1.0 ± 0.6	2.7± 3.0	4.1 ± 2.4	5.9 ± 2.7
Shikimic acid	1	4.6E−13	1.0 ± 0.2	5.2 ± 1.1	5.5 ± 0.9	4.1 ± 0.6
Sucrose (Glc + Fru)	1	9.9E−04	1.0 ± 0.8	3.5 ± 3.5	3.5 ± 3.6	6.9 ± 1.1
Tartaric acid	2	1.3E−03	1.0 ± 0.4	2.0 ± 0.4	1.3 ± 0.6	2.0 ± 0.4
Tyrosine	1	1.5E−02	1.0 ± 0.3	1.2 ± 0.3	1.4 ± 0.2	1.6 ± 0.2

T1: 4 weeks’ treatment; T2: 3 weeks’ treatment; T3: 2 weeks’ treatment. ID level (1: confirmed with analytical standards; 2: confirmed with retention index and mass spectra from NIST and GMD; 3: non-identified metabolite—functional group prediction according to their mass spectra).

#### Experiment 3: MIS growth modeling

The metabolite profile of Experiment 3 was explored similarly to Experiment 2. Firstly, the missing values (155, 5.1%) of the entire metabolite dataset were replaced by 1/5 of the minimal corresponding variables. The resulting matrix contained the relative levels of 70 metabolites/features in 43 samples from six groups, being three sampling points for control and treated samples. These were then log-transformed and explored to assess potential differences in the metabolite profile between groups. As shown in Fig. 10C, the two principal components of the PCA model explained 46.6% of the total variance among samples. The first component (34.4% of the total variance) was the main component responsible for separation between samples from the first/initial sampling point (i.e., Ci and Ti) and those from second/intermediary and third/final sampling points (i.e., Cm, Cf, Tm, and Tf). The biplot of the PCA model is attached as Supplementary Fig. S1B.

The apparent separation between control and treatment on the 2D-PCA plot was confirmed using a strategy similar to that described by Miller et al.^49^. Samples were grouped as control and treatment, and the scores in eight dimensions (91% of the explained variance) were selected for further linear discriminant analysis (LDA). The prediction model using the calculated distances of the eight dimensions showed good fitting (Wilks’ Lambda < 0.001). Classification results revealed that the probability of membership of the control group was higher than 80% for all control samples, while the probability for the treatment group was higher than 95% for all treated samples. LDA also confirmed PC1 as the best predictor based on its higher standardized canonical discriminant function value (1.6), followed by PC2 (1.4). This order was consistent with the order of the structure matrix. We then performed a *t*-test on the score values projected onto PC1 and confirmed significant differences ($p < 0.01$) between control ($-1.40 \pm 1.20$, *n* = 8) and treatment ($0.49 \pm 0.89$, *n* = 23) groups. We also found significant differences on the score values when the control was compared separately with T1, T2, or T3, but no statistically significant differences were found in pairwise comparisons of T1, T2, and T3. The loadings which mainly contribute to the separation along PC1 were proline and serine (higher correlation with control samples), while sucrose and 2-OG were the loadings most highly correlated with treated samples.

We also applied principal component regression using the eight components to define variables that can be used as the best predictors. Using this multiple linear regression model, proline, shikimic acid, and GABA were shown to be in correlation potential with the presence of mechanical stress (Supplementary Fig. S3).

ANOVA (FDR-adjusted $p < 0.05$) showed 34 metabolites with significant differences among groups (Table 4). However, no clear differences between the metabolite profile of control and treated samples was found. As can be seen on the heatmap (Fig. 10D), samples were clustered mainly according to the sampling point/development stage rather than treatment. To confirm these results, differences between control and treatment on each sampling point were explored individually (i.e., Cf vs. Tf, Cm vs. Tm, Ci vs. Ti). No significant differences (*t*-test, $p < 0.05$) were found between control and treated samples at specific sampling points. PCA plots taken individually from samples of each sampling point/development stage also suggested no clear differences in the metabolite profile of samples from the control and treatment groups (Supplementary Fig. S2).

Although no clear differences between control and treatment were found at the final sampling from Experiment 3, some metabolites showed similar response patterns to that of Experiment 2. These metabolites include 2-OG and maltose, which were found at higher levels on control samples in both experiments, as well as GABA and shikimic acid, which were increased in treated samples.

**Table 4:** Metabolites with significant differences (ANOVA, FDR-adjusted $p < 0.05$) on Experiment 3 (greenhouse). Relative levels in relation to control samples obtained on the third/final sampling point (Cf).

Compound	ID level	q-value	Cf	Cm	Ci	Tf	Tm	Ti
2-Oxoglutarate (2-OG)	2	3.20E−05	1.0 ± 0.4	0.2 ± 0.1	3.0 ± 1.6	0.5 ± 0.6	0.2 ± 0.1	2.8 ± 1.1
5-Oxoproline	2	1.00E−12	1.0 ± 0.2	2.5 ± 0.5	0.3 ± 0.1	1.4 ± 0.7	3.0 ± 0.7	0.3 ± 0.1
Alanine	1	1.30E−08	1.0 ± 0.3	1.0 ± 0.4	0.3 ± 0.1	1.1 ± 0.4	1.3 ± 0.4	0.3 ± 0.2
Aspartic acid	1	6.00E−03	1.0 ± 0.3	1.1 ± 0.4	0.6 ± 0.2	1.1 ± 0.5	1.3 ± 0.2	1.0 ± 0.4
Dehydroascorbic acid (DHA)	2	3.40E−06	1.0 ± 0.1	0.9 ± 0.1	0.8 ± 0.1	1.1 ± 0.1	1.0 ± 0.1	0.7 ± 0.1
Fumaric acid	1	6.40E−03	1.0 ± 0.4	0.5 ± 0.1	0.6 ± 0.2	1.4 ± 0.7	0.9 ± 0.4	0.6 ± 0.1
GABA	1	5.20E−11	1.0 ± 0.3	0.4 ± 0.1	0.3 ± 0.1	1.2 ± 0.1	0.6 ± 0.2	0.3 ± 0.1
Glucose	1	2.20E−04	1.0 ± 0.2	1.1 ± 0.1	0.9 ± 0.0	1.2 ± 0.2	1.3 ± 0.2	0.9 ± 0.2
Glycerol	2	1.10E−10	1.0 ± 0.1	0.9 ± 0.1	0.5 ± 0.2	1.2 ± 0.2	1.0 ± 0.1	0.4 ± 0.1
Hexanoic acid	2	5.50E−07	1.0 ± 0.2	0.7 ± 0.1	0.4 ± 0.0	1.2 ± 0.2	0.8 ± 0.2	0.4 ± 0.1
Lactose (Glc + Gal)	1	5.00E−07	1.0 ± 0.3	0.5 ± 0.0	0.3 ± 0.0	1.1 ± 0.3	0.7 ± 0.2	0.3 ± 0.0
Maleic acid	2	4.70E−03	1.0 ± 0.3	1.2 ± 0.5	0.7 ± 0.2	1.5 ± 0.6	1.3 ± 0.5	0.7 ± 0.2
Malic acid	1	2.10E−04	1.0 ± 0.3	0.6 ± 0.2	0.3 ± 0.2	0.9 ± 0.1	0.6 ± 0.1	0.5 ± 0.3
Maltose (Glc + Glc)	2	2.00E−09	1.0 ± 0.1	1.0 ± 0.1	1.2 ± 0.2	1.0 ± 0.0	1.0 ± 0.0	1.2 ± 0.2
Myo-Inositol	2	1.20E−03	1.0 ± 0.1	0.6 ± 0.1	0.8 ± 0.5	1.0 ± 0.1	0.7 ± 0.1	0.5 ± 0.3
NI amine	3	3.40E−06	1.0 ± 0.2	0.8 ± 0.2	0.4 ± 0.1	1.1 ± 0.3	0.9 ± 0.2	0.4 ± 0.1
NI carboxylic acid	3	9.70E−04	1.0 ± 0.2	0.8 ± 0.2	1.7 ± 0.9	0.9 ± 0.2	0.9 ± 0.1	2.1 ± 0.9
NI carboxylic acid	3	6.60E−04	1.0 ± 0.1	0.8 ± 0.1	0.9 ± 0.1	1.1 ± 0.1	0.9 ± 0.1	0.9 ± 0.1
NI carboxylic acid	3	5.20E−04	1.0 ± 0.1	0.9 ± 0.1	0.8 ± 0.2	1.0 ± 0.1	1.0 ± 0.1	0.7 ± 0.1
NI carboxylic acid	3	9.90E−04	1.0 ± 0.1	0.7 ± 0.2	0.6 ± 0.4	1.1 ± 0.2	0.9 ± 0.2	0.5 ± 0.3
NI carboxylic acid	3	1.40E−04	1.0 ± 0.1	0.9 ± 0.1	0.7 ± 0.3	1.0 ± 0.1	1.0 ± 0.1	0.6 ± 0.1
NI carboxylic acid	3	6.70E−03	1.0 ± 0.2	0.8 ± 0.1	0.5 ± 0.1	1.1 ± 0.3	0.8 ± 0.1	0.7 ± 0.3
NI carboxylic acid	3	7.90E−04	1.0 ± 0.1	0.9 ± 0.1	0.8 ± 0.3	1.0 ± 0.1	1.0 ± 0.1	0.6 ± 0.2
NI carboxylic acid	3	9.20E−03	1.0 ± 0.3	0.8 ± 0.1	0.6 ± 0.1	1.1 ± 0.2	0.9 ± 0.1	0.5 ± 0.1
NI carboxylic acid	3	1.10E−03	1.0 ± 0.1	0.8 ± 0.1	0.8 ± 0.3	1.1 ± 0.1	0.9 ± 0.1	0.7 ± 0.2
NI sugar	3	3.20E−05	1.0 ± 0.2	0.8 ± 0.1	0.7 ± 0.2	1.1 ± 0.1	0.9 ± 0.1	0.6 ± 0.1
NI sugar	3	8.40E−08	1.0 ± 0.2	0.9 ± 0.1	0.5 ± 0.2	1.0 ± 0.2	1.0 ± 0.2	0.4 ± 0.2
Nonanoic acid	2	4.70E−03	1.0 ± 0.1	1.0 ± 0.1	1.9 ± 0.6	1.1 ± 0.1	1.0 ± 0.1	2.1 ± 0.7
Palmitic acid	1	1.30E−03	1.0 ± 0.1	0.8 ± 0.1	7.4 ± 7.0	1.2 ± 0.2	0.8 ± 0.1	9.6 ± 6.8
Phosphoric acid	2	2.40E−08	1.0 ± 0.2	0.5 ± 0.1	0.5 ± 0.1	1.0 ± 0.1	0.5 ± 0.1	0.6 ± 0.1
Proline	1	5.40E−06	1.0 ± 0.2	0.7 ± 0.1	0.5 ± 0.1	1.1 ± 0.4	1.0 ± 0.4	0.6 ± 0.1
Serine	1	1.70E−07	1.0 ± 0.1	1.1 ± 0.2	0.4 ± 0.1	1.1 ± 0.4	1.3 ± 0.2	0.4 ± 0.2
Shikimic acid	1	6.70E−03	1.0 ± 0.2	1.4 ± 0.4	0.8 ± 0.2	1.4 ± 0.8	1.7 ± 0.4	0.9 ± 0.4
Succinic acid	1	1.70E−07	1.0 ± 0.1	1.0 ± 0.3	0.4 ± 0.1	1.2 ± 0.4	1.0 ± 0.3	0.5 ± 0.1
Sucrose (Glc + Fru)	1	4.10E−03	1.0 ± 0.3	0.9 ± 0.4	3.0 ± 2.2	1.1 ± 0.1	1.0 ± 0.2	2.8 ± 2.3
Valine	1	1.30E−08	1.0 ± 0.2	0.8 ± 0.1	0.5 ± 0.1	1.0 ± 0.2	1.0 ± 0.2	0.5 ± 0.0

Control and treated samples obtained at the first/initial (Ci, Ti), second/intermediary (Cm, Tm) and third/final (Cf, Tf) sampling points. ID level (1: confirmed with analytical standards; 2: confirmed with retention index and mass spectra from NIST and GMD; 3: non-identified metabolite—functional group prediction according to their mass spectra).

### Elements

#### Evaluation of protocol design and data collection integrity

**Figure 11:**
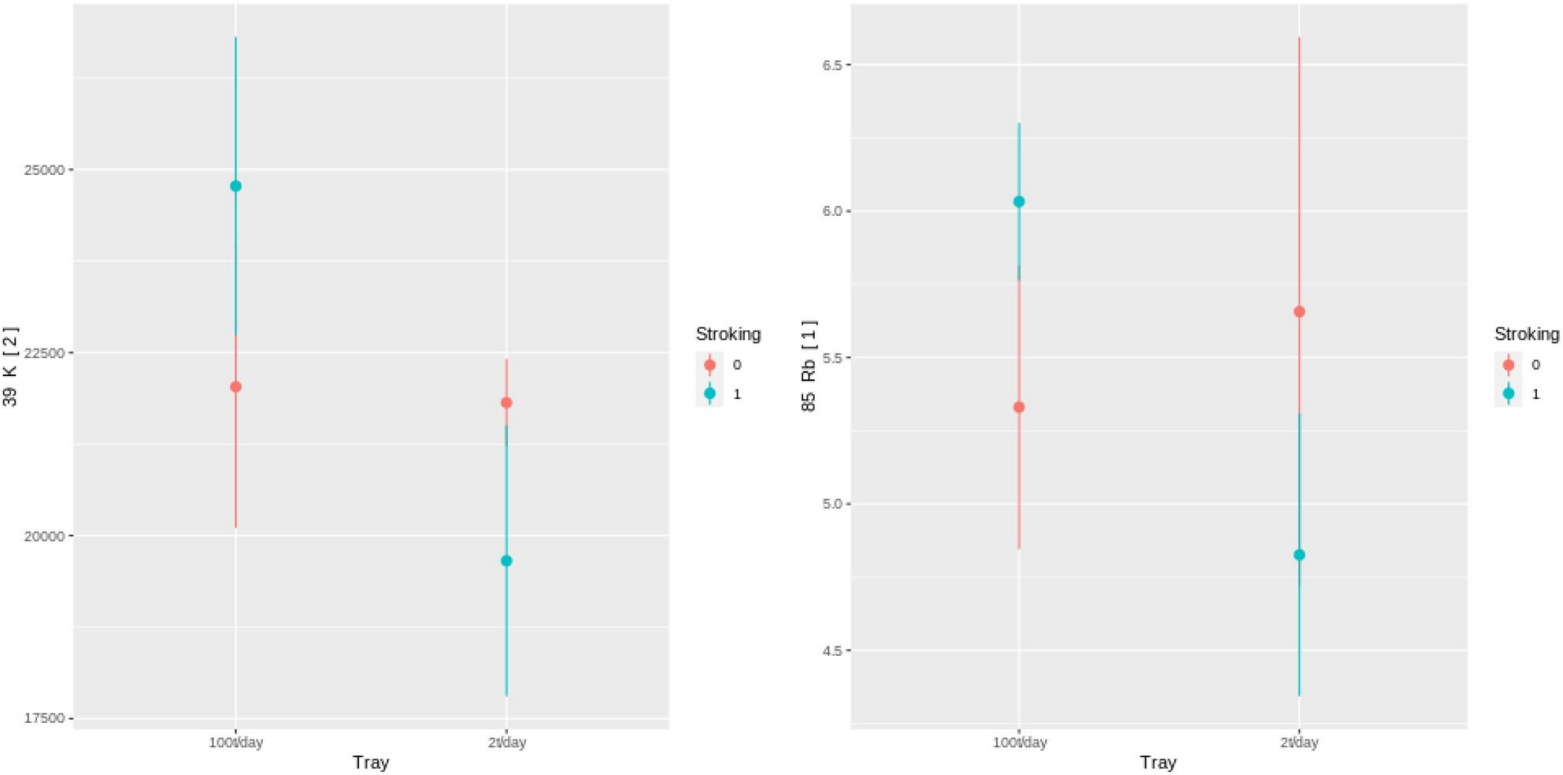
Average levels and 95% interval of K and Rb ($\upmu$ g/g) for the eight collected samples in the two treatment groups (100 t/day and 2 t/day for stroking = 1) and two control groups (stroking = 0).

**Figure 12:**
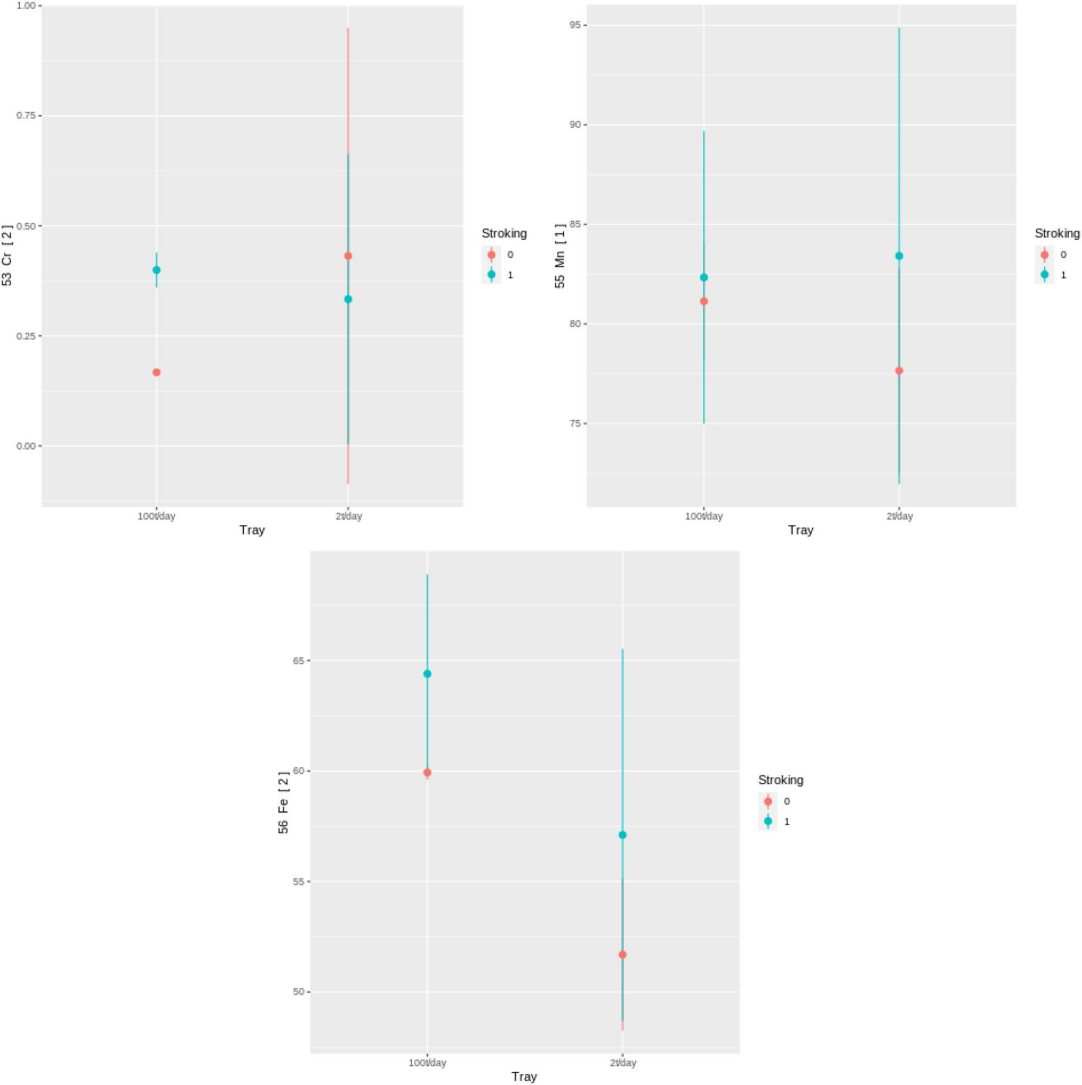
Average levels and 95% interval of Cr, Mn, and Fe ($\upmu$ g/g) for the eight collected samples in the two treatment groups (100 t/day and 2 t/day for stroking = 1) and two control groups (stroking = 0).

**Figure 13:**
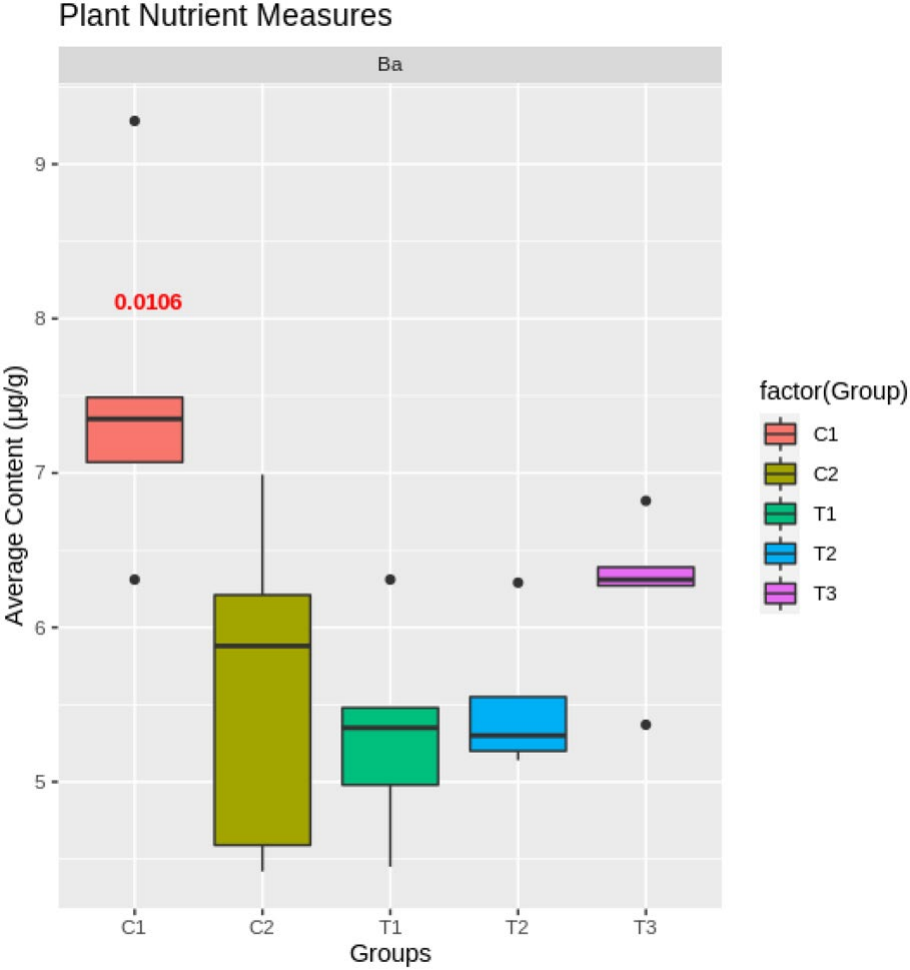
Test-bed distribution of average content of Ba[1] ($\upmu$ g/g) as a function of the treatment group (T1–T3) or control group (C1, C2).

The eight samples collected in Experiment 1 were not subject to a statistical evaluation due to the low number of samples. They were used to perform data collection integrity evaluation. The sample showed large differences between control groups in very few of the collected elements: only Ag, Pb, Li, and Fe. These results, similar to the morphological control group comparison results, suggest that the conditions between the two trays in the first greenhouse experiment are similar. A detailed look into element groups with similar chemical behavior and properties was used to evaluate the treatment’s impact on the plants. Several groups are possible but two were mainly used for the evaluation. In the case of K and Rb, it is clear that they behave similarly, and the increased uptake in plants stroked 100 times/day indicate that the stressed plants increase their uptake of ions from the soil compartment (Fig. 11). The increase in both K and Rb indicates that the increased uptake is not selective for a specific element but is rather only an increased uptake of ions in general due to the induced stress. Similar evidence arose for divalent and trivalent ions such as Cr, Mn, and Fe, for which a similar trend as for K and Rb can be seen (Fig. 12). Comparison of control groups for the data collected in Experiment 3 have shown statistically significant differences for most elements between control groups (for example, BA[1]; Fig. 13). These results, supported by the borderline significance of differences between control groups in the morphological measures of Experiment 2 (reported above), lead us to believe there are other strong influences within the growing bed masking the affects of the mechanical stress induction. As a result, the analysis of the metabolite results collected from the test bed will not be reported.

The comparison of control groups for the data collected in Experiment 3 shows differences in control groups as well. This suggests that, while no visible morphological changes or metabolic changes were found between the control groups as reported above, some differences between the conditions of *Tray1* and *Tray2* were present. Possible explanations for differences in conditions are described in the “Discussion” section of this paper. As a result of these differences all comparisons between groups in subsequent analysis of Experiment 3 are done within each tray.

#### Effect of stroking on element absorption

In Experiment 3, almost all observed elements (Table 5) showed increased average values for plants undergoing a mechanical stress treatment, by 20–250%. The exception was Te [1], showing a reduction of up to 100% in both *Tray1* and *Tray2*. Some of the resulting increase values, specially in *Tray2*, seem to be exceptionally high (Cr and Ni, marked in Table 5), and may potentially be an outlier result. Possible explanations could be their low absolute amount in the sample, making them prone to measurement error. To obtain the exact increase values for these elements, further investigation is required in the form of a repetition of the experiment with more raw material. Nevertheless, these results strongly suggest that mechanical stress induces the absorption of elements by the plant grown in potted greenhouse conditions. In Experiment 2, performed in the test bed, some differences in element absorption can be seen as well, but as mentioned previously, due to the differences between control groups and lack of a paired control group for each treatment group, no conclusions can be drawn from these differences.

**Table 5:** Average elements content increase ($\upmu$g/g and relative %) between mechanically induced plants and their control group in each tray.

Element	Tray1 ($\upmu$g/1 g)	Tray1 (%)	Tray2 ($\upmu$g/1 g)	Tray2 (%)
7 Li [1]	0.55	157.43%	0.32	55.23%
9 Be [1]	0.12	49.84%	0.38	146.81%
23 Na [1]	231.70	266.26%	317.40	165.29%
24 Mg [1]	1907	64.09%	42.50	1.08%
27 Al [1]	33.82	57.08%	154.89	189.97%
39 K [2]	45,812	110.38%	15,934	26.63%
43 Ca [1]	3887.93	21.69%	− 637.81	− 2.53%
51 V [2]	0.05	120.17%	0.58	774.05%
53 Cr [2]	0.20	42.84%	5.01	1099.58%$^{\text {a}}$
55 Mn [1]	11.36	37.91%	24.85	47.39%
56 Fe [2]	29.65	35.04%	362.43	256.86%
59 Co [1]	0.19	115.91%	0.33	89.90%
60 Ni [1]	0.23	194.50%	22.79	6615.83%$^{\text {a}}$
63 Cu [1]	3.10	99.25%	5.36	85.11%
66 Zn [1]	19.60	78.87%	− 1.51	− 2.99%
69 Ga [1]	0.06	38.96%	0.36	178.83%
75 As [2]	0.12	27.79%	0.66	166.04%
82 Se [2]	0.38	22.47%	2.21	153.56%
85 Rb [1]	10.14	148.55%	2.22	20.16%
88 Sr [1]	5.41	29.49%	1.61	6.37%
95 Mo [1]	0.04	13.26%	0.89	153.71%
107 Ag [1]	0.01	43.62%	0.04	146.81%
111 Cd [1]	0.01	16.79%	0.07	164.32%
125 Te [1]	0.00	− 95.22%	− 0.01	− 49.54%
137 Ba [1]	0.68	36.32%	4.93	171.52%
205 Tl [1]	0.00	38.72%	0.02	146.81%
208 Pb [1]	0.05	29.25%	0.81	426.49%
209 Bi [1]	0.33	165.34%	0.08	145.88%
238 U [1]	0.02	110.96%	0.17	517.03%

$^{\text {a}}$Potential outliers due to the very low presence of the element in the sample.

## Discussion

The results we have reported show significant differences in morphological and chemical traits of plants that have undergone mechanical treatment by the robotic systems we designed. They also show how differences in treatment frequency, start and end times, and stage of development affect the plant’s overall response to mechanical stimuli. The morphological changes, specifically the decrease in stem and plant size, is consistent with previously published results^5^, and was shown to be reproducible in two consecutive experiments in greenhouse conditions. This suggests that the protocols identified can be reproduced, with the goal of altering plant morphology. The ability to do so with no chemical intervention, the currently common practice, is of a great importance for developing healthier and more sustainable growing practices. Sampling multiple times through the growing cycle showed no effect on the length of the already developed inter-nodes before the mechanical stress started to be induced, with similar stem prolongation for both control and treatment groups. Repeated samples of the subsequent inter-nodes showed significant increasing differences, not only in absolute inter-node length but also in inter-node development speed. These results suggest that the differences in stem length originate in the development speed of the upper inter-nodes, and therefore support the previously reported results where MIS inhibited plant development. Nevertheless, comparative analysis of leaf weight between treated and untreated groups in Experiment 1, previously reported by us in Kurtser et al.^41^, has shown no statistically significant differences in leaf weight. These results suggest that growth inhibition of the stem does not inhibit the development of the leaves, the part of the basil plant used commercially. This data collection could not be reproduced for the other experiments (Experiments 2–3) outlined in this paper, due to the need to sample the leaves for metabolite and nutrient profiling, affecting the overall leaf weight and development.

The increase in element absorption, as a result of mechanical stress, is correlated with the shorter and sturdier plants developed. These plants seemed to absorb more elements to build resilience against harsh outdoor conditions. To put the increase into perspective, Mg [1] is used as a paradigm as follows. The observed increase of Mg in Tray 1 was $\sim$ 1900 $\upmu$g/g, a 64% increase compared to the control group. Given that the daily recommended intake of Mg by an adult male (United States Institute of Medicine^51^) is around $\pm 400$ mg, 1 g (the equivalent of about 0.5 teaspoon) of stroked basil plants compared to 1 g of controlled basil plants will account for 0.5% of daily intake. Given that basil plants are not considered to be the main source of Mg in the human diet, the difference is rather significant. It is important to note, though, that almost all elements were increased, as well as ones that are generally considered to have negative influence on health like Na (salt). According to the reported results, the observed increase in Na of 230–300 $\upmu$g/g, compared to the control, will also account for an increase of 0.008% of the maximum daily recommended intake of salt, for the same 1 g of dried basil plants.

Metabolite profiling also revealed differences in the metabolism of plants that have undergone mechanical treatment. From a plant physiology perspective, results suggest that treated plants were clearly subjected to stress conditions, as observed by the increased levels of dehydroascorbic acid (DHA) and GABA, particularly in Experiment 2. An increase in the intrinsic levels of DHA suggest either an increase in ascorbate oxidase activity due to oxidative stress, or a decrease in monodehydroascorbate reductase, responsible for regeneration of ascorbic acid, an oxidant scavenger in plants^52^. Therefore, higher DHA levels relate to an increase in the formation of reactive oxygen species (ROS), which might occur in plants due to gentle mechanical sweeping of leaf surfaces^53^. Although an increase in ROS is often related to deleterious effects on plants, studies suggest that ROS are predominantly beneficial to plants—reviewed by Mittler^54^—and necessary for basic biological processes such as cellular proliferation, differentiation, and, to a larger extent, adaptation to distinct environmental conditions. This adaptation hypothesis might explain why plants subjected to 4 weeks’ treatment appeared to have a metabolite profile more similar to that of the control, compared to those plants subjected to 2 or 3 weeks’ treatment.

Treated plants also had up to a two-fold increase in the levels of GABA, which often accumulates in response to abiotic stresses, being an important intermediate of nitrogen metabolism and amino acid biosynthesis in plants^55^. The increase in GABA and DHA in mechanically treated plants was followed by a decrease in 2-oxoglutarate (2-OG) levels. 2-OG is a tricarboxylic acid cycle (TCA) intermediate which have a crucial role in a range of oxidative reactions. Furthermore, 2-OG is considered to be a bridge linking plants’ primary and specialized/secondary metabolism^56^.

Plant stress is interconnected with physiological events needed for adaptation, such as the synthesis of protective compounds for the plants. Notably, many of these protective compounds synthesized by the plant act as functional compounds in humans as well. Therefore, the apparent stress observed in mechanically treated plants can be positive from a nutritional perspective. Supporting such a hypothesis, mechanically induced plants showed up to 20% higher accumulation of methionine, an essential amino acid for humans. Furthermore, the decrease of 2-OG, an intermediary metabolite for plants’ secondary metabolism, indicates a higher metabolic flux on this pathway, which can lead to the formation of flavonoids. This latter class of metabolites is the most common and widely distributed group of phenolic antioxidants in plants. There is considerable evidence from observational population studies linking the consumption of plants rich in flavonoids to health effects, including reduction in the risk of cardiovascular disease^57^. Since the applied two-step derivatization method focused mainly on the analysis of polar metabolites including carboxylic acids, alcohols, and amino acids (i.e., covering mainly compounds associated with primary plant metabolism), this study was not able to identify phenolic compounds arising from secondary plant metabolism. Nevertheless, such changes in 2-OG levels strongly suggest the accumulation of bioactive compounds.

Another important result is the inability to reproduce the same element content and metabolite pattern results in the growing bed setting, despite using the same stroking material and stroking frequency as in the greenhouse experiment. The authors hypothesize that one or several of the following factors could be to blame. First, the growing bed means all plants share the same soil, which in turns means that interactions between roots are likely and that, e.g., root exudates could be evenly distributed throughout the soil compartment. This might explain the differences between control groups, as well as their spatial location close to different regions of soil, and soil element content affected by other stressed plants might be masking the main differences. Second, the watering method used in the greenhouse (bottom watering and removal of water) results in a different availability of free elements and their drainage from the soil, compared to top watering with no drainage in the growing bed. Third, the borderline significant difference between the control groups in morphological measures obtained from the growing bed (Experiment 2), suggests possible differences in growing conditions between the different areas. Despite the plants being placed in the same temperature and humidity conditions, under a professional growing light, the plants were planted very densely and therefore the distribution of light might have been affected by neighboring plants. Finally, the inability to continue mechanical stress induction during nights and weekends (as opposed to continuous, around-the-clock operation in the greenhouse), might have also masked the effect of MIS, with the plants having sufficient time to recover from previously induced stress. Similarly, the differences in element absorption between the control groups in the two trays of Experiment 3 also remains unexplained. In the experiment, we observed metabolomic temporal changes rather than differences between control and treated samples. Such results are expected, since differences in plant developmental stages are expected to overcome differences in acclimation due to mild mechanical stress. We didn’t find differences in the metabolite profile between control and treated samples in the greenhouse experiment. We also didn’t find differences between control and treated samples collected at the same developmental stage (Supplementary Fig. S4). The observation of differences in element absorption between control samples from different trays might explain the lack of within-group homogeneity in the metabolite profile of greenhouse samples once metabolism can be easily affected by small changes in nutrient absorption^58^. An investigation into the hypotheses stated above could be conducted in future work through the collection of soil and water samples for element analysis, but at this stage the authors are not able to report that the MIS effect can be reproduced in growing beds, a closer equivalent to a field-growing crop.

Finally, the two robotic platforms developed have shown the ability to research the different aspects of mechanical stress in different plant-growing conditions. The robotic platform developed in the growing bed shows how the results from the greenhouse setup can be generalized by the use of similar motion frequency and stroking materials to conditions resembling a commercial setting. The commercial-like setup achieved similar results to previously published three-DoF robotic systems^20,21^. The concerns regarding the usage of MIS in growing bed conditions outlined above, including sharing the same soil between control and treatment groups, can be tested by using the robotic system developed in a potted plants. The seven degrees-of-freedom manipulator used in the greenhouse conditions was found to be redundant in the growing bed conditions, and three DoF were sufficient for the current use-case. Despite this fact, the authors do not suggest abandoning the additional degrees of freedom for evaluation of possible stroking motions at this point, as they have shown to produce significantly different results for different motion types. For full commercialization, though, the motion developed from the seven-DoF manipulator can be introduced in a lower-DoF system, as shown in the growing bed conditions. Full commercialization of the methods described above would require system design considerations in the specific greenhouse before any more concrete suggestions can be made.

## Conclusions

This paper presents two robotic platforms aimed towards improving plant quality using precise mechanical stress induction. The use-case for basil growing has been shown to validate previously published results, with significant shortening of overall stem length (by up to 40%) and inter-node distances (by up to 80%) observed for plants treated with a robotic mechanical stress-induction protocol, compared to control groups. The results have also shown an increase in absorption of almost all investigated elements by 20–250%, suggesting that the plants can absorb more nutrients from the soil. Metabolite profiling also revealed that stressed plants have a higher metabolic flux towards the production of secondary metabolites, which are often associated with beneficial effects after human consumption. Results also suggest that shorter periods of stroking close to harvest (e.g., two weeks before harvest) might have a greater impact on plants’ metabolite profiles, compared to longer periods of stroking (e.g., four weeks before harvest), due to plant adaptation.

In this research, we showed how the platforms developed are capable of adjusting the different variables of a mechanical stimulus treatment protocol. The results of the use-case have shown the impact of the adjustment of these variables as part of the cultivation protocol on the final grown product.

As suggested before, the required mechanical stimulation frequency to produce significant results supports the claim that an automated robotic system is required. The inability to find conclusive results in the growing bed setup might also be connected to the reduced stimulus setup, but this assumption is to be tested. Further development of the experimental protocols and their validation is required, before adaptation of the protocols by commercial growers. The platforms described can be used for future recreation and validation of the results, and possible extension to larger-scale experiments. The platforms can also be used for development of new protocols, varying additional aspects of mechanical stress induction.

## Supplementary Information

Supplementary Figures.
