# Supplementary material for: Development of novel robotic platforms for mechanical stress induction, and their effects on plant morphology, elements, and metabolism
Source: Sci Rep. 2021 Dec 13;11:23876. doi: 10.1038/s41598-021-02581-9 (PMC8669031; doi:10.1038/s41598-021-02581-9)
Supplement: Supplementary file 1 — Supplementary Figures. [file 41598_2021_2581_MOESM1_ESM.pdf]

Supplementary Figures

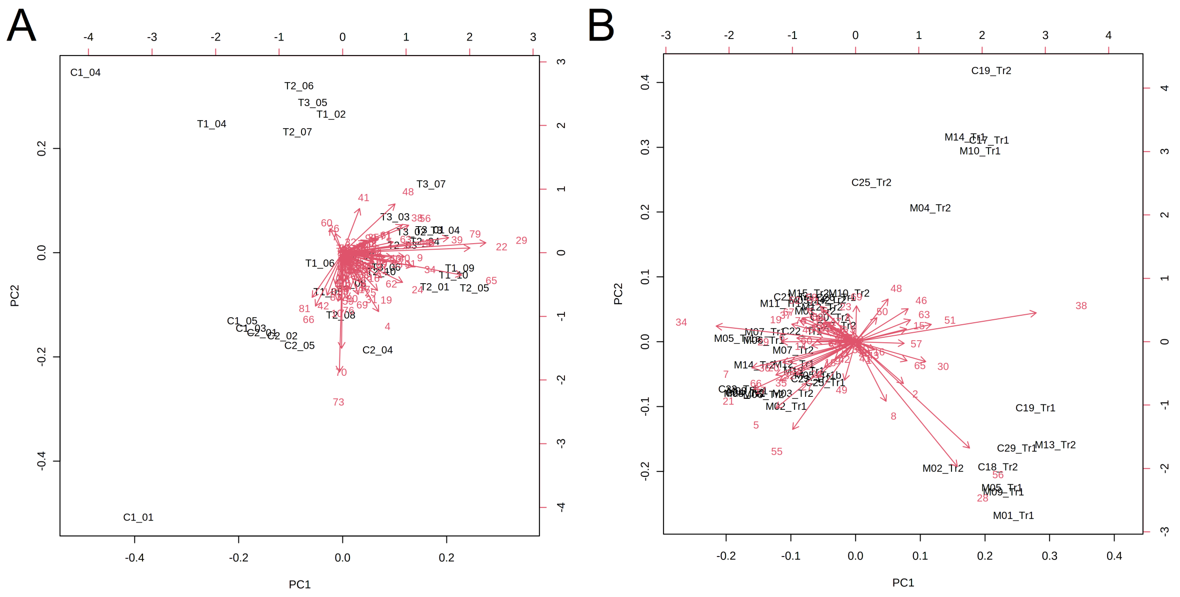

Figure S.1: PCA biplot for control vs. treatment.

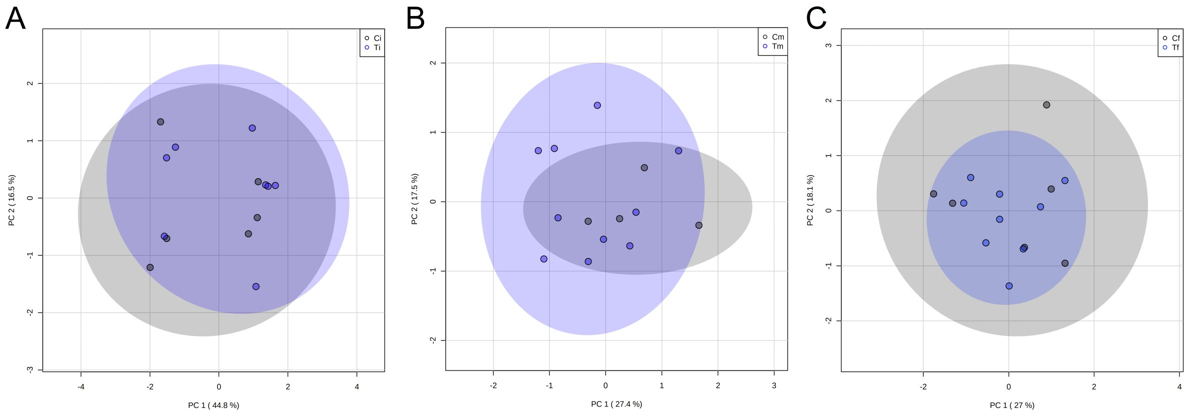

Figure S.2: PCA loading plot for control and treated samples collected at (A) first/initial (Ci, Ti), (B) second/intermediary (Cm, Tm) and (C) third/final (Cf, Tf) sampling points.

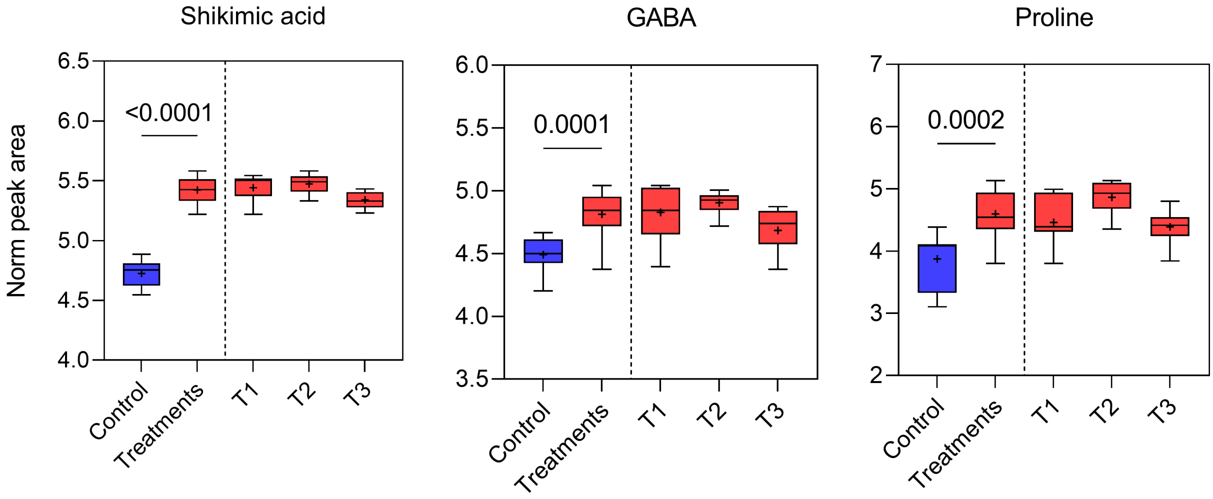

Figure S.3: Relative levels of shikimic acid, GABA and proline in control and treated samples. Numbers above bars represent the p-value (t-test, control vs. treatment).

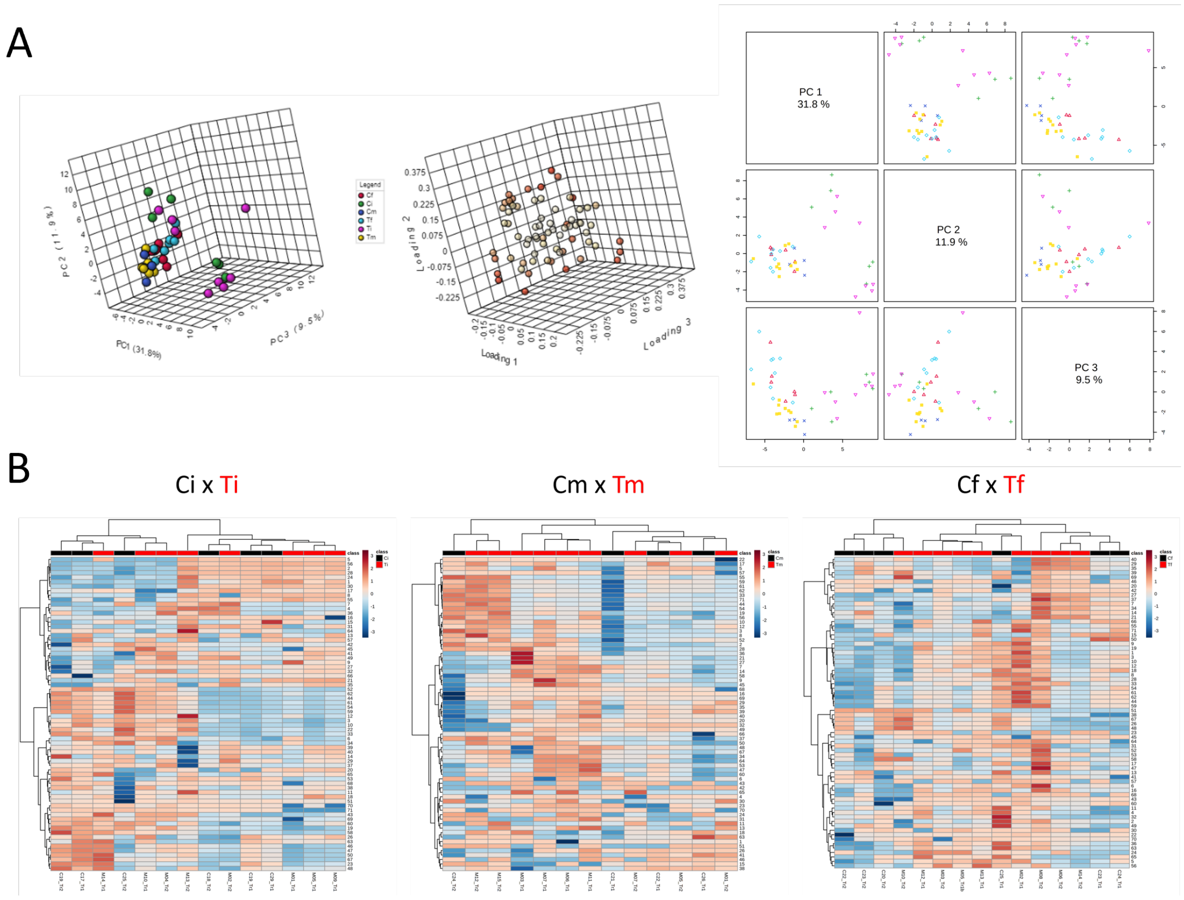

Figure S.4: (A) 3D-PCA plot of Experiment 3 and (B) heatmap of control and treated samples at distinct developmental stages (Euclidean distances, Ward method for agglomeration). Control and treated samples obtained at the first/initial (Ci, Ti), second/intermediary (Cm, Tm) and third/final (Cf, Tf) sampling points. Figure generated using the open source program *Metaboanalyst*[50].
